# A randomised fractional factorial screening experiment to predict effective features of audit and feedback

**DOI:** 10.1186/s13012-022-01208-5

**Published:** 2022-05-26

**Authors:** Alexandra Wright-Hughes, Thomas A. Willis, Stephanie Wilson, Ana Weller, Fabiana Lorencatto, Mohamed Althaf, Valentine Seymour, Amanda J. Farrin, Jillian Francis, Jamie Brehaut, Noah Ivers, Sarah L. Alderson, Benjamin C. Brown, Richard G. Feltbower, Chris P. Gale, Simon J. Stanworth, Suzanne Hartley, Heather Colquhoun, Justin Presseau, Rebecca Walwyn, Robbie Foy

**Affiliations:** 1 Leeds Institute of Clinical Trials Research, University of Leeds, Leeds, UK; 2 Leeds Institute of Health Sciences, University of Leeds, Leeds, UK; 3 Centre for Human-Computer Interaction Design, City, University of London, London, UK; 4 Centre for Behaviour Change, University College London, London, UK; 5 School of Health Sciences, University of Melbourne, Melbourne, Australia; 6 Ottawa Hospital Research Institute, Ottawa, Canada; 7 School of Epidemiology and Public Health, University of Ottawa, Ottawa, Canada; 8 Department of Family and Community Medicine, Womens College Hospital, University of Toronto, Toronto, Canada; 9 Centre for Health Informatics, University of Manchester, Manchester, UK; 10 Centre for Primary Care, University of Manchester, Manchester, UK; 11 Leeds Institute for Data Analytics, School of Medicine, University of Leeds, Leeds, UK; 12 Leeds Institute of Cardiovascular and Metabolic Medicine, University of Leeds, Leeds, UK; 13 Department of Cardiology, Leeds Teaching Hospitals NHS Trust, Leeds, UK; 14 Transfusion Medicine, NHS Blood and Transplant (NHSBT), Oxford, UK; 15 Department of Haematology, Oxford University Hospitals NHS Foundation Trust, Oxford, UK; 16 Radcliffe Department of Medicine, University of Oxford, Oxford, UK; 17 NIHR Oxford Biomedical Research Centre, Oxford, UK; 18 Department of Occupational Science and Occupational Therapy, University of Toronto, Toronto, Canada

**Keywords:** Audit and feedback, Randomised fractional factorial experiment, MOST, Behaviour change

## Abstract

**Background:**

Audit and feedback aims to improve patient care by comparing healthcare performance against explicit standards. It is used to monitor and improve patient care, including through National Clinical Audit (NCA) programmes in the UK. Variability in effectiveness of audit and feedback is attributed to intervention design; separate randomised trials to address multiple questions about how to optimise effectiveness would be inefficient. We evaluated different feedback modifications to identify leading candidates for further “real-world” evaluation.

**Methods:**

Using an online fractional factorial screening experiment, we randomised recipients of feedback from five UK NCAs to different combinations of six feedback modifications applied within an audit report excerpt: use *effective comparators*, provide *multimodal feedback*, recommend *specific actions*, provide *optional detail*, incorporate the *patient voice*, and minimise *cognitive load*. Outcomes, assessed immediately after exposure to the online modifications, included intention to enact audit standards (primary outcome, ranked on a scale of −3 to +3, tailored to the NCA), comprehension, user experience, and engagement.

**Results:**

We randomised 1241 participants (clinicians, managers, and audit staff) between April and October 2019. Inappropriate repeated participant completion occurred; we conservatively excluded participant entries during the relevant period, leaving a primary analysis population of 638 (51.4%) participants.

None of the six feedback modifications had an independent effect on intention across the five NCAs. We observed both synergistic and antagonistic effects across outcomes when modifications were combined; the specific NCA and whether recipients had a clinical role had dominant influences on outcome, and there was an antagonistic interaction between *multimodal feedback* and *optional detail*. Among clinical participants, predicted intention ranged from 1.22 (95% confidence interval 0.72, 1.72) for the least effective combination in which *multimodal feedback*, *optional detail*, and *reduced cognitive load* were applied within the audit report, up to 2.40 (95% *CI* 1.88, 2.93) for the most effective combination including *multimodal feedback*, *specific actions*, *patient voice*, and *reduced cognitive load*.

**Conclusion:**

Potentially important synergistic and antagonistic effects were identified across combinations of feedback modifications, audit programmes, and recipients, suggesting that feedback designers must explicitly consider how different features of feedback may interact to achieve (or undermine) the desired effects.

**Trial registration:**

International Standard Randomised Controlled Trial Number: ISRCTN41584028

**Supplementary Information:**

The online version contains supplementary material available at 10.1186/s13012-022-01208-5.

Contributions to the literature

- There are many potential ways to optimise the effects of audit and feedback, but conducting multiple separate randomised trials to address individual effectiveness research questions would be inefficient.
- In an online screening experiment, we randomised participants from five national clinical audits to different combinations of six feedback modifications applied within an audit report excerpt to identify feedback modifications and their combinations worthy of further real-world evaluation.
- Whilst none of six feedback modifications had independent effects on intention to enact audit standards (a proxy outcome theorised to influence actual clinical behaviour), we observed significant synergistic and antagonistic interactions between modifications, participant role, and national clinical audit.
- Our fractional factorial design uniquely provides direct information on the effects of both individual feedback modifications and their interactions, demonstrating an empirical approach to optimising multicomponent interventions.

## Background

Audit and feedback, as a component of quality improvement, aims to improve the uptake of recommended practice by reviewing clinical performance against explicit standards and directing action towards areas not meeting those standards [1]. Around 60 national clinical audit (NCA) programmes in the UK [2] inform service improvement across priorities such as diabetes, stroke, and cancer.

Whilst a number of components which may enhance effectiveness have been identified (e.g. providing feedback more than once), feedback effectiveness remains difficult to predict [1, 3, 4]. Rigorous evaluation methods, including randomised trials, can establish the relative effectiveness of alternative feedback components. Given there are many potential ways of delivering feedback components (e.g. timing, comparators, display characteristics), with or without co-interventions (e.g. educational meetings, computerised reminders), addressing all would require a prohibitive number of head-to-head trials and would not allow investigation of interactions between interventions and their components. More efficient methods are needed to prioritise which feedback components to study and to take forward to definitive trials.

The multiphase optimization strategy (MOST) offers a methodological approach for building, optimising, and evaluating multicomponent interventions [5, 6]. MOST comprises three steps: *preparation*, laying the groundwork for optimisation by conceptualising and piloting components; *optimisation*, conducting trials to identify the most promising single or combined intervention components; and *evaluation*, a definitive randomised trial to assess intervention effectiveness. Earlier implementation studies have used a similar approach to define the most promising “active ingredients” for further study [7, 8], including experiments that systematically vary components of an intervention within a randomised controlled design in a manner that simulates a real situation as much as possible. Interim endpoints (e.g. behavioural intention, behavioural simulation) are measured rather than actual behaviour or healthcare outcomes. A key mechanism of effect of audit and feedback interventions is that they operate by increasing recipients’ intention to enact desired changes in accordance with the audit standards.

We undertook the first and second steps of MOST to develop and investigate the single and combined effects of different feedback components (hereby referred to as “feedback modifications”). We began with a set of 15 theory-informed suggestions for effective feedback, identified through expert interviews, systematic reviews, and our own experience with providing, evaluating, and receiving practice feedback [3]. These suggestions were grouped under the nature of the desired action (e.g. improving the specificity of recommendations for action), the nature of the data available for feedback (e.g. providing more rapid or multiple feedback), feedback display (e.g. minimising unnecessary cognitive workload for recipients), and delivery of feedback (e.g. addressing credibility of information). We considered and added a further suggestion (incorporating the patient voice) in response to current policy drives to involve patients and members of the public more in health service organisation and delivery [2].

We used a structured consensus process, involving audit and feedback developers, recipients and researchers, and public representatives, to select the following six feedback modifications as high priority for investigation in an online fractional factorial screening experiment [9]: *effective comparators*, *multimodal feedback*, *specific actions*, *optional detail*, *patient voice*, and *cognitive load*. The consensus panel guided our selection based on the need for further research, likely feasibility of adoption by national clinical audits, feasibility of delivery within an online experiment, and user acceptability. We then engaged professionals typically involved in developing or targeted by NCAs in three rounds of user-centred design to develop and apply the modifications within an audit report excerpt and design a web portal for the online experiment.

In the second stage of a MOST, reported here, we used a randomised fractional factorial screening design, to investigate and optimise the most promising single and combined effects of the six modifications on interim outcomes. We chose a factorial design to allow all six modifications and their interactions to be investigated simultaneously. By randomising participants to multiple modification conditions, all participants contribute to the evaluation of each effect, with a reduced sample size compared to an equivalent evaluation in multiple or multi-arm multistage adaptive trials, which would in any case detect different estimands (i.e. simple effects rather than main and interaction effects) to those of interest here.

## Methods

### Design overview

We conducted an online, fractional factorial screening experiment. Six modifications to feedback (Table 1; see also Additional file 1) were each operationalised in two versions (ON with the modification applied, OFF without modification) and applied within audit report excerpts for five different NCAs. We randomised participants to receive one of 32 combinations of the modifications, stratified by NCA. After viewing the audit excerpt, participants completed a short questionnaire, to generate all study outcomes. This study is reported as per the CONSORT guideline for randomised trials [10].

**Table 1:** The six feedback modifications selected in our online fractional factorial screening experiment

Modification description	Modification ON vs OFF
***A. Effective comparators*** Feedback is typically given in the context of a comparator. Select comparators according to their ability to change or reinforce the desired behaviour	ON when showing the top 25% nationally as the comparator OFF when showing the mean average
***B. Multimodal feedback*** Present feedback in different ways to help recipients develop a more memorable mental model of the information presented, allow interaction with the feedback in a way that best suits them, and reinforce memory by repetition	ON if the performance result text was accompanied by a graphical display of performance data OFF when the graphical display was absent
***C. Specific actions*** Specify desired behaviour to facilitate intentions to perform that behaviour and enhance the likelihood of subsequent action	ON if the feedback suggested specific recommendations for action (i.e. who needs to do what, differently, with or to whom, where and when) OFF when such recommendations were absent
***D. Optional detail*** Provide short, actionable messages with optional information available for interested recipients. Feedback credibility can be enhanced if recipients are able to ‘drill down’ to better understand their data	ON if short messages with clickable, expanding links to explanatory detail were included OFF when these links were absent
***E. Patient voice*** Explicitly link patient experience to audit standards to highlight the importance of providing high-quality care and hence increase motivation to improve practice	ON when a box including a photograph of a fictional patient was added, with a quotation describing their experience of care related to the associated audit standard OFF when these were absent
***F. Cognitive load*** Minimise the effort required to process information by prioritising key messages, reducing the amount of data presented, improving readability, and reducing visual clutter	ON when distracting detail was minimised OFF if additional general text not directly related to the audit standard and feedback on other audit standards was added

### Setting and participants

We collaborated with five UK NCAs covering a range of clinical priorities: the Myocardial Ischaemia National Audit Project (MINAP) [11], National Comparative Audit of Blood Transfusion (NCABT), Paediatric Intensive Care Audit Network (PICANet), and Trauma Audit Research Network (TARN) in secondary care and the National Diabetes Audit (NDA) in primary care. The NCABT, MINAP, and TARN each covered more than 150 National Health Service (NHS) trusts in England alone. PICANet included 34 paediatric intensive care units, and the NDA covered all (approximately) 7500 general practices in England.

Each NCA emailed invitations containing the link to the online experiment to their distribution lists of feedback recipients, i.e. clinicians, managers, nurses, and commissioners; all were eligible to participate. Prior to experiment entry, participants were required to confirm informed consent. On completing the experiment, participants were offered the opportunity to view evidence-based guidance on how to improve their own audit and feedback practice. Participants were also offered a £25 voucher and certificate of completion. Email addresses provided for voucher and certificate requests were not linked to experiment data to preserve anonymity.

After opening to recruitment, we identified a serious breach of study integrity involving inappropriate repeated participant completion of the experiment linked to a single general practice in order to claim multiple £25 vouchers for completion. This occurred within a 5-day period subsequently defined as the “contamination period”. We therefore temporarily closed the experiment to enhance security. Additional experiment entry criteria, applied prior to randomisation, required participants to provide NHS or Health and Social Care Northern Ireland email addresses. These were validated to confirm that participants had not previously completed the experiment and to prevent those who had, from proceeding; email addresses remained unlinked to experiment data to retain anonymity.

### Intervention

Following consent, participants selected the audit relevant to them, before indicating their role and organisation. Participants were then randomised to be presented with one of 32 versions of the excerpt of an audit report comprising different combinations of the six modifications (each ON or OFF). Participants were informed that the excerpt contained simulated but realistic data.

The audit excerpts followed a basic template (Additional file 1). The page was titled with the relevant audit (e.g. “National Diabetes Audit Report”) and a statement that the data were collected in 2018. The excerpt showed an audit standard (e.g. “Patients with type 2 diabetes whose HbA1c level is 58 mmol/mol or above after 6 months with single-drug treatment are offered dual therapy”) and the result (e.g. “Our practice achieved this standard of care for 86% (318/370) of patients”). NCA collaborators advised on the selection of audit standards to help ensure experiment participants perceived them as valid and credible [12]. The remaining content depended on which combination of the six feedback modifications participants were randomised to (Table 1).

### Outcomes

The primary outcome was participant intention to adhere to an audit-specific standard (Table 2). Intention has a known, if limited, ability to predict behaviour that may inform intervention development and early evaluation [13–15].

**Table 2:** NCA standards contributing to experiment outcomes

*NCA*	*Key standard selected*
NCABT	Clinical staff should prescribe tranexamic acid for surgical patients expected to have moderate or more significant blood loss unless contraindicated
NDA	Patients with type 2 diabetes whose HbA1c level is 58 mmol/mol (7.5%) or above after 6 months with single-drug treatment are offered dual therapy
MINAP	Adults with non-ST-segment-election myocardial infarction or unstable angina who have an intermediate or higher risk of future adverse cardiovascular events are offered coronary angiography (with follow-on percutaneous coronary intervention if indicated) within 72 h of first admission to hospital
PICANet	Minimise the number of unplanned extubations for paediatric intensive care patients per 1000 days of invasive ventilation
TARN	Patients who have had urgent 3D imaging for major trauma should have a provisional written radiology report within 60 min of the scan

We aimed to minimise unintended “loading” of responses of intention due to social desirability bias by presenting the target behaviour in the context of other behaviours that would be appropriate, including the introductory statement, “Considering the time and resources available to you and other clinical priorities …”, and anchored items over “the next three months”.

The primary outcome measured intention as the mean value across three items beginning with the stem statements, “I intend”, “I want”, and “I expect”. Each item was followed by the appropriate audit standard, e.g. “Over the next three months, I *[intend/want/expect]* to ensure that our patients with type 2 diabetes whose HbA1c level is 58mmol/mol or above following 6 months with single-drug treatment are offered dual therapy”. Responses to each item followed a 7-point Likert scale and were scored −3 (completely disagree) through to +3 (completely agree). Previous testing of these stems indicated that they measure the same concept, with Cronbach’s alpha values above 0.9 [16].

Secondary outcomes, mainly assessed on a −3 to +3 Likert scale, comprised the following:

- *Proximal intention* evaluating participants’ intention to undertake other actions in response to feedback: bring the audit result to the attention of colleagues, set goals, formulate an action plan, and review personal performance in relation to the audit standard.
- *Comprehension* using a single item (“I found the information in this audit report excerpt easy to understand”) adapted from the Website Evaluation Questionnaire [17].
- *User experience* using the mean value of the positively worded two-item lite version of the Usability Metric for User Experience questionnaire [18–20]: “This audit report excerpt met my information needs”, and “This online audit report excerpt was easy to use”.
- *User engagement* using the length of time (in seconds) spent on and the number of “clicks” within the audit report excerpt.

### Data collection

After viewing the audit excerpt, participants completed a 12-item questionnaire displayed within the experiment. We recorded the time spent on the excerpt and the questionnaire and the number of “clicks” on the audit page.

### Statistical considerations

#### Experimental design

A full factorial design would require 2^6^ = 64 combinations of the six modifications. We chose a half fraction of the full design, i.e. 32 combinations, to provide a more efficient design to identify the vital few (significant) factors from the trivial many (screening).

We generated our balanced and orthogonal half fractional factorial design [5, 21], denoted ${2}_{VI}^{6-1}$, using the defining relation *I* = ABCDEF, design generator *F* = ABCDE, and effect coding with each level of the six modifications coded as −1 (OFF) and +1 (ON).

The trade-off using the half, rather than the full design, is the introduction of aliasing (confounding) in model effects. Under the half fraction with six factors, all effects are aliased; main effects are aliased with 5-way interactions, 2-way interactions aliased with 4-way interactions, and 3-way interactions form aliased pairs. Under the sparsity of effects principle [22], in which a system is usually dominated by main effects and low-order interactions, we assume negligible four-way and higher-order interactions and attribute any effects to the main effects and lower-order interactions.

Although the full factorial design, with no aliasing, would have allowed estimation of higher order effects, this would have required increased resource to implement and verify all 64 combinations across the five NCAs. Considering existing knowledge and assumptions about interactions, we therefore chose the half fraction to minimise the number of combinations required whilst allowing estimation of all main effects and 2-way interactions of modifications. We considered but discounted use of the quarter fraction as further aliasing would have compromised the interpretation of 2-way interactions. The full design and alias structure can be found in Additional file 1.

#### Randomisation and masking

Participants were allocated to one of the 32 combinations of the six feedback modifications, with equal allocation using block randomisation, stratified by NCA. The design was replicated in blocks of the 32 combinations, each partitioned into two blocks of 16 using the alias pair ABF = CDE, to ensure modifications were balanced (each modification has the same number of participants at each level) and orthogonal (sum of the product of any two or more modifications is 0) within each block of 16 participants. A statistician (AWH) prepared the randomisation lists, which were programmed (MA) into the website. Remaining study personnel remained blind to allocation. Participants were, by nature of the experiment, exposed to the randomised audit excerpts but not informed of their allocation.

#### Sample size

Assuming similar effects of each modification across NCA and role, 500 participants across the five NCAs provided 90% power to detect small-to-moderate main effects (0.3 SDs) for each modification using a two-sided 5% significance *t*-test. Due to the use of effect coding for each modification, all else being equal, there is equal power for detecting interaction effects (of any order, irrespective of aliasing) of the same magnitude as the main effects. Any antagonistic interactions would reduce the magnitude of main effects; this sample size provided approximately 80% and 70% power to detect reduced main effects of 0.25 SDs and 0.22 SDs, respectively. No allowance for loss to follow-up was required, as data were collected at one time point. As this was a screening experiment, the aim was to identify potentially important effects for further evaluation (by ruling out unimportant effects). No allowance was made for multiplicity because false positives are identified through further experimentation. Detection of promising effects was based on the use of Pareto plots, where a split was made between potentially important and unimportant effects.

Recruitment was permitted to exceed the 500 participant target, up to a maximum of 1200 participants (480 participants per NCA, 15 replications of the 32 combinations of modifications), to increase the power to evaluate potential interaction effects within available resources. We originally planned a 4-month recruitment period.

### Statistical analysis

#### Populations

We defined two modified intention-to-treat (ITT) populations. The primary population excluded all participants recruited during the “contamination period” over which repeated participant completion took place. A secondary population excluded participants who completed the experiment questionnaire in < 20 s for sensitivity analyses (based on the distribution of questionnaire completion times, Additional file 2). This cutoff was chosen to provide a more inclusive population compared to the primary population, aiming to retain valid and unique participants during the contamination period whilst removing those most likely to be duplicative participants who completed the questionnaire in an unfeasible time.

#### General considerations

Statistical analyses described in a pre-specified plan, approved by the independent statistician from our project steering committee, were conducted in SAS version 9.4 (SAS Institute Inc., Cary, NC). An overall two-sided 5% significance level was used unless otherwise stated.

#### Analytical approach

To identify and screen for potentially active modifications, we included the six experimental modifications and covariates as independent variables in multivariable linear regression models (using maximum likelihood estimation) with dependent variables for the primary outcome of intention and secondary outcomes of proximal intention, comprehension, and user experience. We used summary statistics to explore the secondary outcome of user engagement.

The pre-specified covariates were as follows:

- NCA: MINAP, NCABT, NDA, PICANet, and TARN. The NCA with the largest number of randomised participants (NDA) formed the reference category.
- Randomised design block: block 1 and block 2, using effect (−1, +1) coding.
- Role: Clinical (allied health professional, fully trained doctor, nurse or nurse specialist, training doctor) and non-clinical (manager, audit and administrative staff). Clinical roles formed the reference category.

We assumed a continuous distribution for all outcomes. We explored the distribution of outcomes using descriptive statistics and graphical display, model diagnostics to check validity of statistical modelling. Although outcomes were collected on a 7-point Likert scale, model diagnostics from the linear models were satisfactory, and this approach was considered more appropriate than alternatives including loss of power from dichotomising the outcome or increased complexity from modelling the data using ordinal regression.

We used effect coding for each modification to ensure parameter estimates, and their interactions provided the main effect (rather than simple effects); that is, the effect averaged across all combinations of levels of the other modifications.

Analysis used a multistage approach for each outcome. Stage 1 used available complete data to identify the most promising modifications and interactions. Stage 2 applied the resulting model using the primary population with multiply imputed missing data.

#### Stage 1 — complete case analysis

We tested whether an “initial” model, including modification main effects and two-way interactions alongside covariates, was adequate using the lack-of-fit test [23]. Where lack of fit was observed, we included additional interactions in a “full” model using stepwise selection based on a 15% significance level of the *F* statistic, respecting the hierarchy of effects and checking consistency with Akaike’s Information Criteria and Bayesian Information Criteria.

Based on the Pareto principle that a small number of parameters account for a large portion of the effect, we identified the most promising parameters from the “full” model by ranking their absolute standardised effect sizes in Pareto plots [5]. A final “parsimonious” model was then obtained using backward selection (based on 15% significance level of the *F* statistic) to simplify the model whilst retaining NCA, randomised design block, and promising parameters identified via the Pareto plot.

#### Stage 2 — ITT analysis

We applied stage 1 models to the primary population with multiply imputed missing data using the fully conditional specification predictive mean matching method [24].

A single missing data model generated 50 imputations across all outcomes using predictors: outcome, NCA, role, NCA*role interaction, modification main effects, and two- and three-way interactions. We applied further interactions, between modification main effects and two-way interactions with NCA and with role, where model convergence allowed. We calculated parameter estimates, associated standard errors, and *p*-values using Rubin’s rules [25].

We compared Pareto plots to ensure the inclusion of appropriate parameters. Where there were differences in parameters meeting the threshold for inclusion, we included parameters identified in either stage in the final “parsimonious” model.

Results present the stage 2 final “parsimonious” models and predicted plots to illustrate the direction and strength of identified main effects and interactions (Additional file 3).

#### Sensitivity analyses

Sensitivity analysis explored the impact of the inappropriate repeated participant completion by repeating the analysis of the primary outcome using available complete data in the secondary population (excluding participants completing the questionnaire in < 20 s) as compared to the primary population (excluding participants within the contamination period).

## Results

### Recruitment

**Fig. 1:**
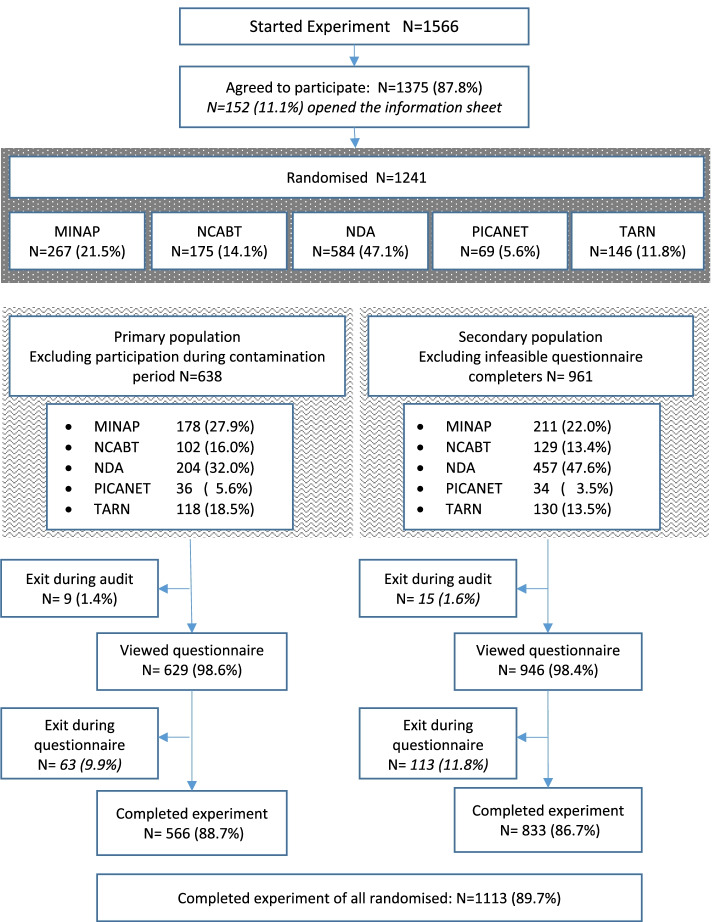
Experiment summary — participant flow

A total of 1241 randomisations were carried out across two recruitment phases (Fig. 1), 967 (77.9%) from 10th to 30th April 2019 and 274 (22.1%) from 5th September to 18th October 2019. The primary population comprised 638 (51.4%) randomisations and excluded all 603 (48.6%) from the 5-day “contamination period” within recruitment phase 1, the highest proportion of which occurred for the NDA (65.1%). The secondary population comprised 961 (77.4%) randomisations, excluding 280 (22.6%) where questionnaires were completed in less than 20 s. All subsequent results relate to the primary population. Additional file 2 presents comparative summaries for the secondary population alongside figures demonstrating that the properties of the randomisation were largely unaffected within the primary and secondary populations.

### Participant characteristics

Participation across NCAs comprised the following: 204 (32.0%) NDA participants, 178 (27.9%) MINAP, 118 (18.5%) TARN, 102 (16%) NCABT, and 36 (5.6%) PICANet (Table 3). Most participants were from hospital trusts (64.9%) or general practices (29.6%). Over 90% of MINAP, NCABT, PICANet, and TARN participants were from hospital trusts, whereas almost 90% of NDA participants were from general practice. Over half (55.2%) reported having clinical roles, 27.3% management roles, and 17.6% audit or administrative roles. Almost 90% of NCABT participants had clinical roles, compared to around half in MINAP, PICANet, and TARN and a third in the NDA.

**Table 3:** Participant characteristics, modifications, and experiment completion

	Participant completed experiment		
	Yes (***n*** = 566)	No. (***n***=72)	Total (***n*** = 638)
**NCA**			
MINAP	158 (27.9%)	20 (27.8%)	178 (27.9%)
NCABT	93 (16.4%)	9 (12.5%)	102 (16.0%)
NDA	172 (30.4%)	32 (44.4%)	204 (32.0%)
PICANet	33 (5.8%)	3 (4.2%)	36 (5.6%)
TARN	110 (19.4%)	8 (11.1%)	118 (18.5%)
**Role**			
Allied health professional	39 (6.9%)	6 (8.3%)	45 (7.1%)
Nurse or nurse specialist	156 (27.6%)	13 (18.1%)	169 (26.5%)
Fully trained doctor	128 (22.6%)	6 (8.3%)	134 (21.0%)
Training doctor	3 (0.5%)	1 (1.4%)	4 (0.6%)
Manager	141 (24.9%)	33 (45.8%)	174 (27.3%)
Audit and admin	99 (17.5%)	13 (18.1%)	112 (17.6%)
**Organisation**			
Commissioning	24 (4.2%)	3 (4.2%)	27 (4.2%)
Community healthcare trust	5 (0.9%)	3 (4.2%)	8 (1.3%)
General practice	160 (28.3%)	29 (40.3%)	189 (29.6%)
Hospital trust	377 (66.6%)	37 (51.4%)	414 (64.9%)
**Seconds on audit report**			
Missing	0	9	9
Median (IQR)	68.5 (33, 138.5)	45.0 (23.5, 110.5)	66.5 (31, 136)
**N. clicks on audit report**			
Missing	0	9	9
Mean (SD)	2.3 (5.34)	1.8 (1.50)	2.2 (5.09)
Median (IQR)	1.0 (1.0, 2.0)	1.0 (1.0, 2.0)	1.0 (1.0, 2.0)
**Seconds on questionnaire**			
Missing	72		72
Median (IQR)	159 (97.5, 255.5)		159 (97.5, 255.5)
**> 20 s**			
Yes	545 (96.3%)		545 (96.3%)
No	21 (3.7%)		21 (3.7%)
**A:*Effective comparators***			
On	292 (51.6%)	33 (45.8%)	325 (50.9%)
Off	274 (48.4%)	39 (54.2%)	313 (49.1%)
**B:*Multimodal feedback***			
On	289 (51.1%)	31 (43.1%)	320 (50.2%)
Off	277 (48.9%)	41 (56.9%)	318 (49.8%)
**C:*Specific actions***			
On	284 (50.2%)	34 (47.2%)	318 (49.8%)
Off	282 (49.8%)	38 (52.8%)	320 (50.2%)
**D:*Optional detail***			
On	273 (48.2%)	39 (54.2%)	312 (48.9%)
Off	293 (51.8%)	33 (45.8%)	326 (51.1%)
**E:*Patient voice***			
On	280 (49.5%)	40 (55.6%)	320 (50.2%)
Off	286 (50.5%)	32 (44.4%)	318 (49.8%)
**F:*Cognitive load***			
On	278 (49.1%)	39 (54.2%)	317 (49.7%)
Off	288 (50.9%)	33 (45.8%)	321 (50.3%)

*SD*, standard deviation; *IQR*, interquartile range

### Randomisation

A similar number and proportion of participants were randomised to each of the 32 combinations of the six modifications (ON or OFF) within and across NCAs (Table 3).

### Experiment completion

A total of 566 (88.7%) participants completed the experiment (Table 3). Non-completers comprised a greater proportion of participants in the NDA, in manager roles, and in general practice, compared to completers. Participants spent a median of 66.5 s (*IQR* 31 to 136) viewing the audit excerpt and 159 s (*IQR* 97.5 to 255.5) completing the questionnaire.

### Outcomes

**Fig. 2:**
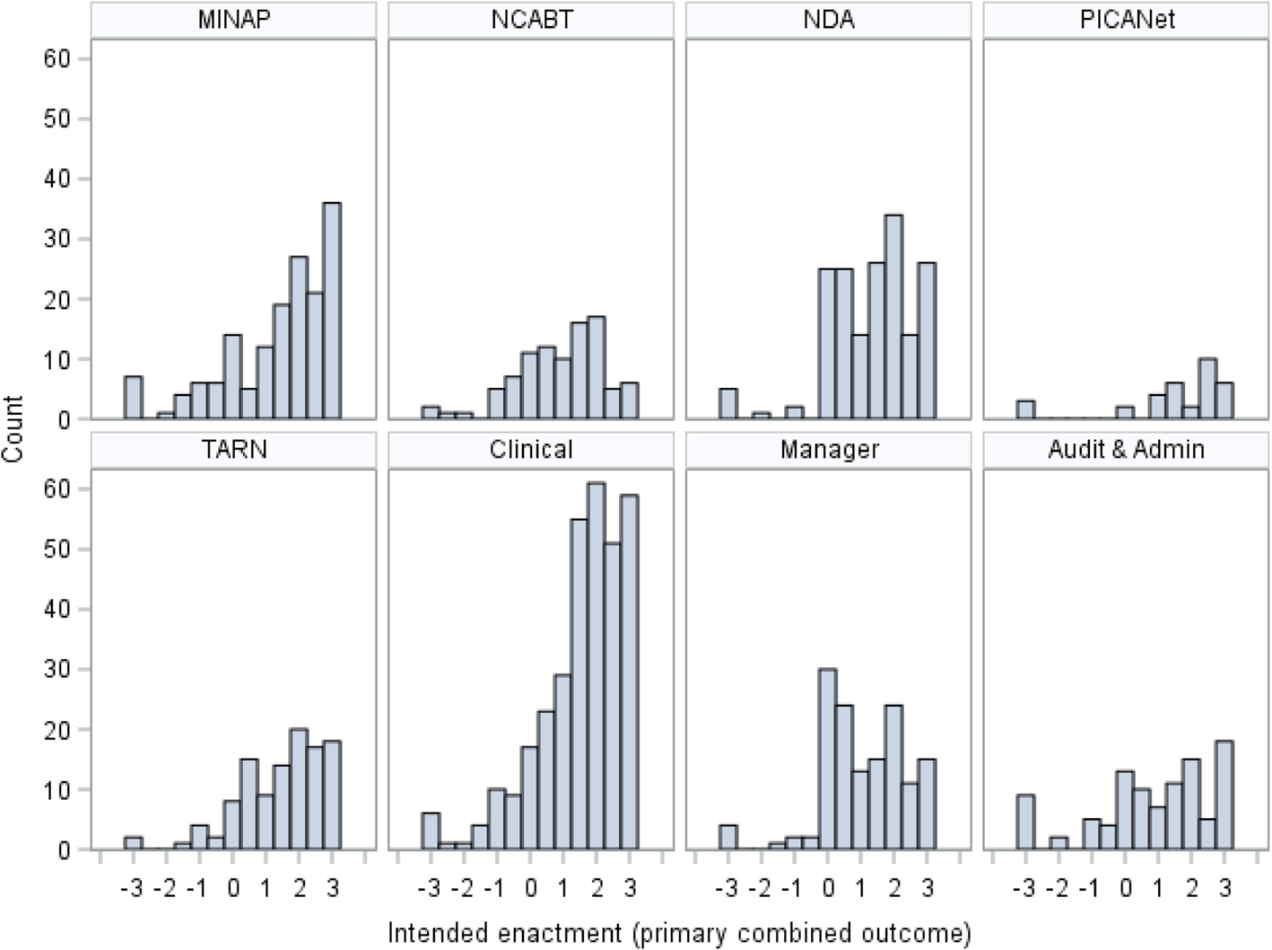
Distribution of the primary outcome by NCA and role

**Fig. 3:**
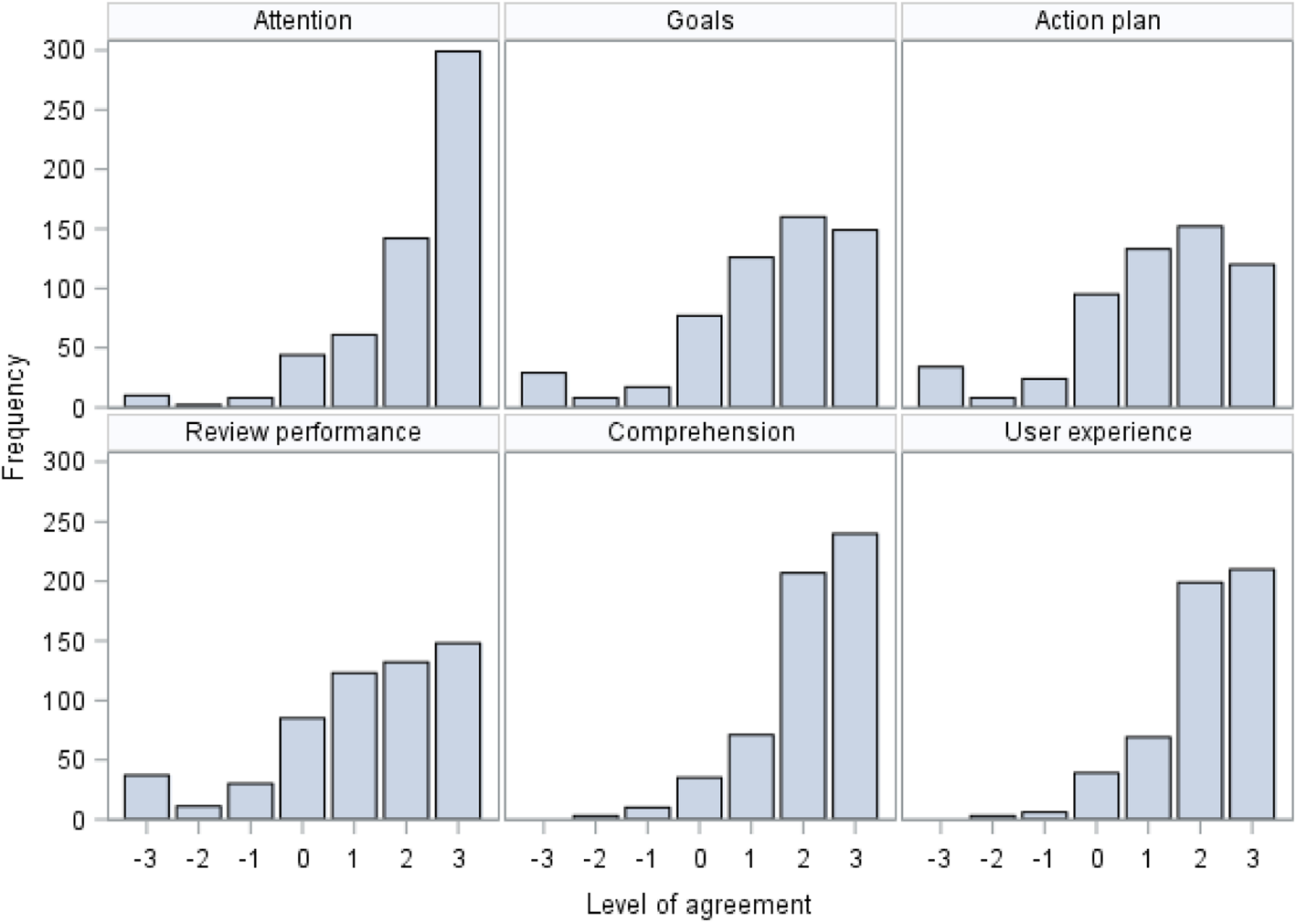
Distribution of secondary outcomes

Response distributions varied across outcomes, and negative skew was present across all outcomes due to ceiling effects (Figs. 2 and 3). Responses for primary outcome components “I intend”, “I want”, and “I expect” were fairly consistent. A Cronbach’s alpha of 0.85, and pairwise correlation coefficients, suggested adequate internal consistency and reliability. The distribution of the primary outcome varied across the NCAs, with a more similar distribution across the NDA, TARN, and MINAP, whilst fewer NCABT participants reported the highest levels of intention. Clinical participants reported higher levels of intention compared to non-clinical participants. Diagnostic plots from stage 1 analysis indicated that residuals were sufficiently normally distributed and homoscedastic with respect to fitted values to retain continuous outcomes.

### Modification effects — primary outcome

**Fig. 4:**
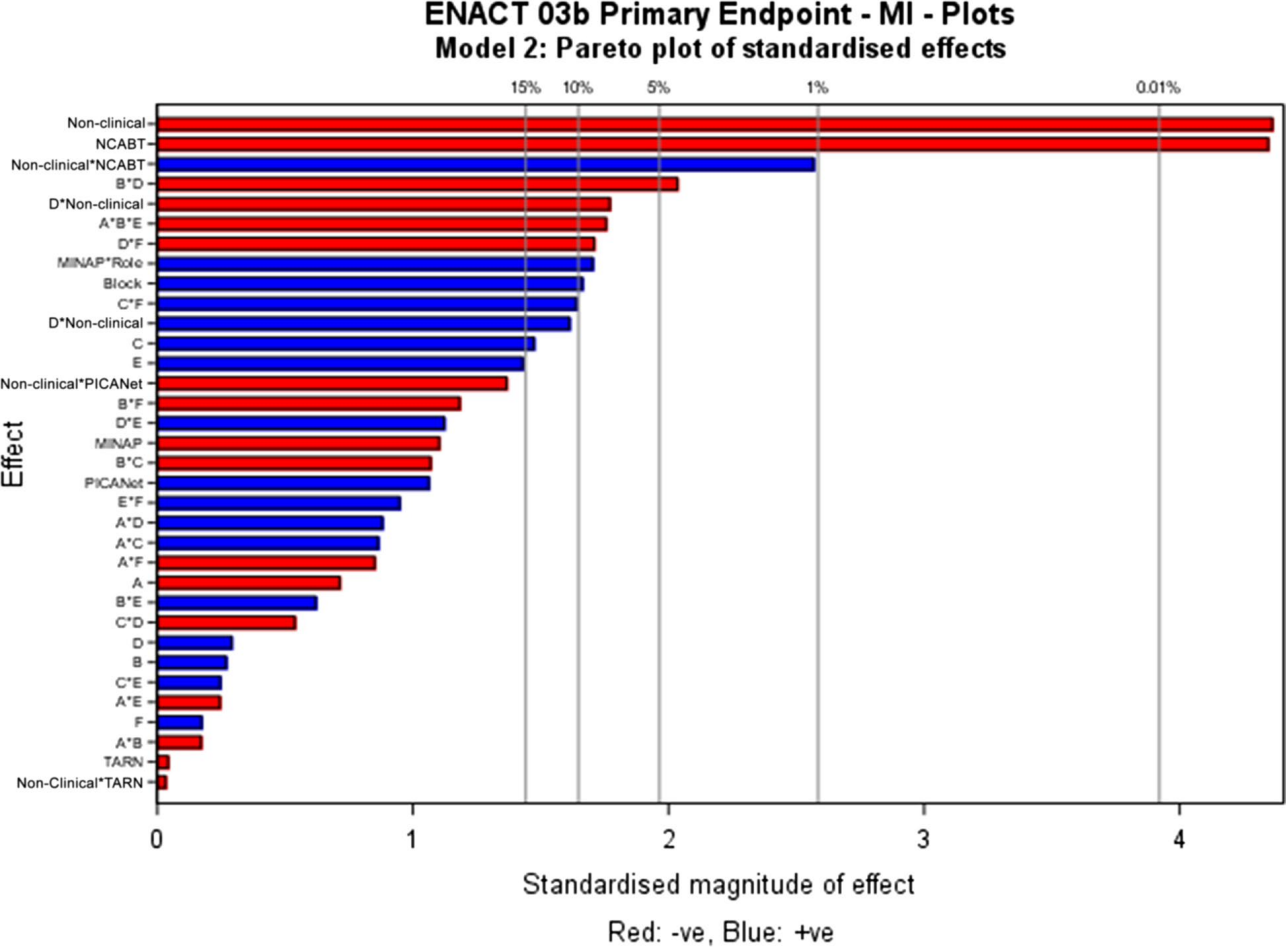
Primary outcome: Pareto plot of standardised effects (primary outcome, stage-2 full model)

No modifications had an independent effect on intention (Table 4). NCA and role had the greatest influence on intention, followed by a role-dependent interaction between *multimodal feedback* and *optional detail* (Fig. 4).

**Table 4:** Parameter estimates for parsimonious models across outcomes*

Parameter	Primary outcome: intention Est (*p*-value)	Proximal intention				Comprehension Est (*p*-value)	User experience Est (*p*-value)
		Attention Est (*p*-value)	Goals Est (*p*-value)	Action plan Est (*p*-value)	Review performance Est (*p*-value)		
**Intercept**	*1.829 (< 0.001)*	*1.812 (< 0.001)*	*1.738 (< 0.001)*	*1.608 (< 0.001)*	*1.335 (< 0.001)*	*2.011 (< 0.001)*	*1.870 (< 0.001)*
**Block**	*0.09 (0.107)*	**−***0.082 (0.120)*	*0.026 (0.681)*	**−***0.017 (0.799)*	**−***0.093 (0.176)*	**−***0.036 (0.390)*	**−***0.042 (0.310)*
**NCA (vs NDA)**							
MINAP	**−***0.211 (0.317)*	**0.521 (< 0.001)**	**−**0.445 (0.069)	**−0.482 (0.049)**	*0.089 (0.607)*	*0.140 (0.203)*	*0.090 (0.407)*
NCABT	**−0.893 (< 0.001)**	*0.253 (0.115)*	**−0.638 (0.006)**	**−0.603 (0.011)**	**−0.565 (0.008)**	*0.135 (0.292)*	*0.118 (0.356)*
PICANet	*0.361 (0.270)*	**0.755 (0.002)**	*0.164 (0.657)*	**−***0.263 (0.491)*	*0.186 (0.548)*	*0.312 (0.110)*	*0.247 (0.206)*
TARN	**−***0.003 (0.989)*	**0.304 (0.050)**	*0.005 (0.984)*	**−***0.275 (0.291)*	*0.246 (0.208)*	*0.022 (0.858)*	*0.014 (0.908)*
**Non-clinical** (vs clinical)	**−0.867 (< 0.001)**		**−0.489 (0.030)**	**−0.571 (0.015)**	**−0.290 (0.043)**		
***A:****Effective comparator*	**−***0.038 (0.498)*	*0.015 (0.778)*	*0.082 (0.190)*	**−***0.018 (0.837)*	**−***0.019 (0.784)*	**−0.091 (0.029)**	**−0.087 (0.036)**
***B:****Multimodal feedback*	*0.018 (0.807)*	*0.016 (0.766)*	**−***0.061 (0.575)*	**−***0.064 (0.313)*	**−***0.052 (0.436)*	*0.054 (0.198)*	*0.048 (0.251)*
***C:****Specific actions*	*0.082 (0.141)*	**−***0.044 (0.400)*	*0.065 (0.285)*	*0.075 (0.240)*	0.118 (0.075)		*0.017 (0.679)*
***D:****Optional detail*	*0.017 (0.816)*		*0.050 (0.415)*	*0.051 (0.434)*	*0.093 (0.174)*	*0.022 (0.603)*	*0.056 (0.176)*
***E:****Patient voice*	*0.078 (0.161)*		*0.059 (0.343)*	*0.064 (0.327)*	*0.088 (0.201)*		**−***0.057 (0.172)*
***F:****Cognitive load*	*0.008 (0.890)*	**0.126 (0.016)**	*0.049 (0.656)*	*0.044 (0.708)*	*0.042 (0.533)*	**0.103 (0.014)**	
A * B	**−***0.011 (0.844)*	*0.083 (0.111)*	*0.081 (0.190)*	*0.073 (0.256)*	0.115 (0.089)		
A * C			**−***0.041 (0.500)*	**−***0.080 (0.210)*			
A * D			*0.069 (0.266)*	*0.095 (0.132)*		0.078 (0.068)	**0.085 (0.043)**
A * E	**−***0.014 (0.798)*		**−***0.046 (0.458)*	**−***0.044 (0.496)*	**−***0.096 (0.153)*		
A * F			**−***0.025 (0.696)*	**−***0.032 (0.616)*			
B * C		**−***0.078 (0.138)*					
B * D	**−0.112 (0.047)**		**−**0.114 (0.067)			**−0.114 (0.008)**	**−0.115 (0.006)**
B * E	*0.035 (0.537)*		*0.013 (0.832)*	*0.026 (0.690)*			
B * F		0.087 (0.089)	*0.057 (0.607)*		**−**0.119 (0.085)		
C * D				**−**0.107 (0.099)	**−**0.119 (0.078)		
C * E							*0.066 (0.116)*
C * F	0.093 (0.09)		*0.059 (0.338)*	*0.050 (0.438)*			
D * E			*0.045 (0.469)*				**0.085 (0.039)**
D * F	**−**0.093 (0.089)				**−**0.123 (0.065)	**−**0.073 (0.079)	
E * F			**−***0.008 (0.894)*				
**Additional interactions**							
A * B * E = C * D * F	**−**0.101 (0.072)		**−**0.121 (0.053)	**−0.137 (0.033)**			
A * C * F = B * D * E			*0.099 (0.109)*	**0.159 (0.015)**			
A * D * E = B * C * F			*0.096 (0.136)*				
A * E * F = B * C * D			**−***0.092 (0.132)*				
Non-clinical * MINAP	*0.453 (0.117)*		**0.721 (0.030)**	**0.695 (0.040)**			
Non-clinical * NCABT	**1.312 (0.011)**		*0.817 (0.146)*	*0.829 (0.160)*			
Non-clinical * PICA	**−***0.783 (0.141)*		**−1.360 (0.021)**	**−***0.708 (0.248)*			
Non-clinical * TARN	**−***0.017 (0.959)*		**−***0.310 (0.406)*	**−***0.135 (0.728)*			
A * Non-clinical				*0.185 (0.160)*			
B * Non-clinical	**−**0.196 (0.083)						
D * Non-clinical	0.195 (0.087)						
B * NCABT			*0.218 (0.247)*				
B * MINAP			**−***0.101 (0.526)*				
B * PICANet			**0.607 (0.035)**				
B * TARN			**−***0.052 (0.775)*				
F * NCABT			**−***0.135 (0.477)*	*0.038 (0.846)*			
F * MINAP			**−***0.094 (0.561)*	**−***0.022 (0.894)*			
F * PICANet			**−***0.080 (0.782)*	**−***0.461 (0.132)*			
F * TARN			**0.357 (0.049)**	**0.407 (0.033)**			
B * F * NCABT			**−***0.170 (0.372)*				
B *F * MINAP			**−0.400 (0.013)**				
B *F * PICANet			**0.569 (0.046)**				
B *F * TARN			**−***0.036 (0.840)*				
**Parameter estimates**							
**• +ve/−ve:** positive or negative effect on outcome **•** Modification interactions + estimate = synergistic/**−**ve estimate = antagonistic							

*The columns identify promising detected effects for each outcome, whilst rows identify consistent effects identified across outcomes. Blank cells represent parameters not included in the model. Parameter estimates are all on the same scale of −3 “completely disagree” to +3 “completely agree”. The model intercept represents the overall predicted mean outcome in the NDA and clinical recipient reference groups, averaged across all possible combinations of modifications. Parameter estimates for NCA and role represent the deviation from the predicted mean outcome for the alternative audit and non-clinical recipients. Positive estimates represent an improvement in outcome compared to the reference NDA and clinical recipients, whereas a negative parameter estimate represents a detrimental effect on outcome. Positive parameter estimates for the main effect of each modification represent an improvement in outcome when the modification is ON (+1) and a negative effect on outcome when the modification is OFF (−1). Conversely, negative parameter estimates represent a negative effect on outcome when the modification is ON (+1) and an improvement in outcome when the modification is OFF (−1). Parameter estimates for interactions between modifications represent the additional deviation from the predicted mean outcome

Intention was lower for non-clinical than clinical participants (−0.867, *SE* = 0.200, *p* < 0.001) in the NDA with similar effects observed within MINAP, PICANet, and TARN. This effect was not observed within the NCABT: intention was lower for clinical NCABT participants than clinical participants in the NDA (−0.893, *SE* = 0.206, *p* < 0.001) and other NCAs (Additional file 3, Fig. A3.1).

There was evidence of an antagonistic interaction between *multimodal feedback* and *optional detail* (−0.112, *SE* = 0.056, *p* = 0.047); intention was lower when both were applied (or not) and higher when only one or the other was applied (Fig. A3.2). In non-clinical participants, there was weak evidence of a negative effect of *multimodal feedback* (−0.196, *SE* = 0.113, *p* = 0.083), and a positive effect of *optional detail* (0.195, *SE* = 0.114, *p* = 0.087), with intention optimised when *optional detail*, not *multimodal feedback*, was provided.

**Fig. 5:**
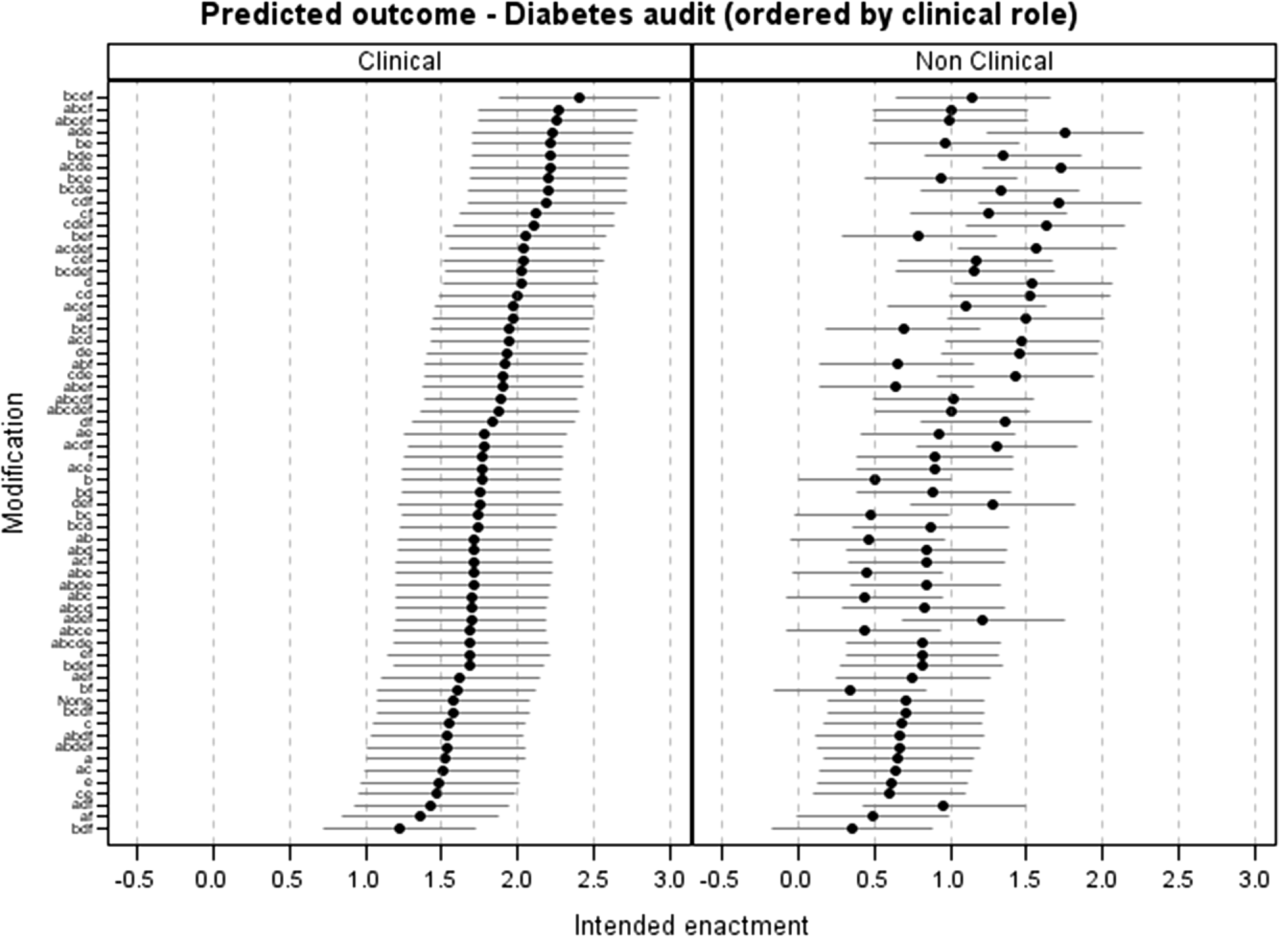
Predicted intention for modification combinations ordered by clinical recipients (results/estimates presented for the NDA only; however, relative effectiveness of modification combinations is consistent across NCAs)

Figure 5 presents predicted intention (on a scale of −3 to +3) of all modification combinations for clinical and non-clinical NDA recipients. The most effective combination in clinical participants across NCAs included *multimodal feedback*, *specific actions*, *patient voice*, and *reduced cognitive load*, with predicted intention of 2.40 (95% *CI* 1.88, 2.93) in the NDA. However, including *multimodal feedback* and *reducing cognitive load* resulted in the least effective combination of modifications when *optional detail* was also provided, with predicted intention of 1.22 (95% *CI* 0.72, 1.72).

Additional file 3 provides further figures relating to the primary analysis and modification effects across outcomes.

### Modification effects — secondary outcomes

#### NCA and role

NCA and role had similar dominant influences on secondary outcomes of proximal intention (Fig. A3.2).

Non-clinical (compared to clinical) participants had lower intention to set goals (−0.489, *SE* = 0.226, *p* = 0.030), create an action plan (−0.571, *SE* = 0.235, *p* = 0.015), and review performance (−0.29, *SE* = 0.143, *p* = 0.043). Interactions with NCA mitigated this effect within MINAP for intention to set goals (MINAP −0.445, *SE* = 0.245, *p* = 0.069; NonClinical*MINAP, 0.721, *SE* = 0.332, *p* = 0.030) and to set an action plan (MINAP −0.482, *SE* = 0.245, *p* = 0.049; NonClinical*MINAP, 0.695, *SE* = 0.339, *p* = 0.040) and accentuated the effect in PICANet (NonClinical*PICANet, −1.360, *SE* = 0.590, *p* = 0.021) on intention to set goals.

As per the primary outcome, reduced intention within clinical NCABT (compared to clinical NDA) participants also mitigated the difference between roles for intention to set goals (NCABT, −0.638, *SE* = 0.234, *p* = 0.006), to set an action plan (NCABT, −0.603, *SE* = 0.238, *p* = 0.011), and to review performance (NCABT, −0.565, *SE* = 0.213, *p* = 0.008). There was no evidence of a difference in intention to bring the audit to the attention of colleagues according to role; however, intention was lowest among participants from the NDA. There was no evidence of a difference in comprehension or user experience according to NCA or role.

#### Modifications: effective comparators and effective comparators*optional detail

An overall average effect of *effective comparators* reduced how easily participants understood the audit report (−0.091, *SE* = 0.041, *p* = 0.029) and their overall user experience (−0.087, *SE* = 0.041, *p* = 0.036). Good to weak evidence of a synergistic interaction between the *comparator* and *optional detail* for these outcomes, comprehension (0.078, *SE* = 0.043, *p* = 0.068), and overall user experience (0.085, *SE* = 0.042, *p* = 0.043), meant the negative comparator effect was not present when *optional detail* was provided (Fig. A3.3).

#### Modifications: cognitive load, multimodal feedback*cognitive load, and optional detail*cognitive load

The overall average effect of reducing *cognitive load* improved intention to bring the report to the attention of colleagues (0.126, *SE* = 0.052, *p* = 0.016, Fig. A3.4) and comprehension (0.103, *SE* = 0.042, *p* = 0.014, Fig. A3.5). There was weak evidence of a synergistic interaction between *cognitive load* and *multimodal feedback* (0.087, *SE* = 0.051, *p* = 0.089, Fig. A3.4), with greater intention to bring the report to the attention of colleagues when *multimodal feedback* was also provided and similar intention otherwise.

A similar synergistic interaction was detected for PICANet participants on intention to set goals (0.569, *SE* = 0.285, *p* = 0.046, Fig. A3.6). The opposite antagonist effect was present for MINAP participants (−0.4, *SE* = 0.162, *p* = 0.013), and a weak antagonistic effect was detected for intention to review performance (−0.119, *SE* = 0.069, *p* = 0.085, Fig. A3.4) across NCAs. 

In TARN participants, reduced *cognitive load* also improved intention to set goals (0.357, *SE* = 0.182, *p* = 0.049, Fig. A3.4) and an action plan (0.407, *SE* = 0.191, *p* = 0.033, Fig. A3.6).

Across all NCAs, there was consistent but weak evidence that reducing *cognitive load* without providing *optional detail* improved comprehension (−0.073, *SE* = 0.042, *p* = 0.079, Fig. A3.5). A similar antagonistic interaction was detected on the primary outcome (−0.093, *SE* = 0.055, *p* = 0.089, Fig. A3.5) and intention to review performance (−0.123, *SE* = 0.066, *p* = 0.065, Fig. A3.5).

#### Modifications multimodal feedback*optional detail

Alongside the primary outcome, there was evidence (Fig. A3.2) of an antagonistic interaction between *multimodal feedback* and *optional detail* on secondary outcomes comprehension (−0.114, *SE* = 0.043, *p* = 0.008) and user experience (−0.115, *SE* = 0.042, *p* = 0.006) and weak evidence for intention to set goals (−0.114, *SE* = 0.062, *p* = 0.067). Outcomes were generally improved when only one of the modifications was applied; however, the intention to set goals was optimised in PICANet participants when *multimodal feedback* was included and not *optional detail* (0.607, *SE* = 0.288, *p* = 0.035).

#### Modifications: optional detail*patient voic﻿e

There was a synergistic interaction between *optional detail* and *patient voice* on user experience such that including *patient voice* without *optional detail* reduced user experience (0.085, *SE* = 0.041, *p* = 0.039, Fig. A3.7).

#### Further modification effects

Two three-way interactions between modifications were also identified across a number of outcomes (Table 4); however, given our fractional factorial design, these effects are confounded by aliased interactions.

#### User engagement

As anticipated, median time spent on the audit tended to be higher when each of the modifications was on, with the exception of *reduced cognitive load* (Fig. A3.8). We observed the greatest number of clicks on the audit excerpt when *optional detail* was on compared to off (Fig. A3.9).

### Sensitivity analysis

Consistency in results using the secondary population, excluding participants who completed the experiment questionnaire in < 20 s, compared to the primary population, excluding the contamination period, is outlined below and further in Additional file 4. The primary outcome, intention, was dominated by the effects of NCA and role to a greater degree in the secondary as compared to the primary population. The dependent effects of *multimodal feedback* with role and *optional detail*, and *optional detail* with *cognitive load* , were broadly consistent across populations. In contrast to our primary analysis, sensitivity analysis using the secondary population identified an additional overall positive effect for *effective comparators* (0.125, *SE* = 0.066, *p* = 0.060) dependent on NCA (−0.246, *SE* = 0.139, *p* = 0.077; −0.302, SE = 0.135, *p* = 0.026).

## Discussion

In an online experiment involving five NCA programmes, none of six feedback modifications independently increased intention to enact audit standards across clinical and non-clinical recipients. However, potentially important synergistic and antagonistic effects were observed when feedback modifications were combined, as well as dominant influences of NCA programme and recipient role. Whilst modification effects were generally small (< 0.1 on a scale of −3 to +3), their combined cumulative effect showed more substantial heterogeneity. Predicted intention for the primary outcome, in clinical participants in the NDA, ranged from 1.22 (95% *CI* 0.72, 1.72) for the least effective combination including *multimodal feedback*, *optional detail*, and *reduced cognitive load* to 2.40 (95% *CI* 1.88, 2.93) for the most effective combination including *multimodal feedback*, *specific actions*, *patient voice*, and *reduced cognitive load*.

Our findings should be considered in the light of Clinical Performance Feedback Intervention Theory (CP-FIT) [26]. This theory specifies steps in the feedback cycle: choosing standards of clinical performance against which care is measured (goal setting); collection and analysis of clinical performance data (data collection and analysis); communication of the measured clinical performance to health professionals (feedback); reception, comprehension, and acceptance of this by the recipient (interaction, perception, and acceptance, respectively); planned behavioural responses to feedback (intention and behaviour); and changes to patient care (clinical performance improvement). A further step of verification may occur between perception and acceptance where recipients interrogate the data underlying their feedback. CP-FIT proposes that feedback, recipient, and context variables operate via a range of mechanisms (e.g. credibility of feedback, social influence) to determine success or failure of the feedback cycle.

Our six feedback modifications and study outcomes mainly focused on perception and intention, although our modifications also targeted interaction, acceptance, verification, and behaviour to lesser extents. Only *reduced cognitive load* alone had positive effects on perception, and *effective comparator* had negative effects. Other feedback modifications’ effects were conditional on interactions, some of which had intuitive explanations. Providing *optional detail* and *multimodal feedback* both entails giving additional information to audit recipients; combining their overlapping functions led to intention being less than the sum of their parts (i.e. an antagonistic interaction). Other interactions were difficult to explain, if not counterintuitive, such as both synergistic and antagonistic interactions between *multimodal feedback* and *cognitive load* for different outcomes, NCAs, and roles. Such a range of findings reflect the exploratory nature of this screening experiment, which aimed to detect the most promising signals of effects for further study.

Our findings suggest that the recipient and context variables of CP-FIT, which approximated to role and NCA in our study, have greater influences on feedback effectiveness than single feedback modifications. Participation in the NCABT was associated with lower intention relative to the NDA as was having a non-clinical role, with the exception of NCABT non-clinical participants. These variations may reflect differences in audit organisation and specialty engagement with audit programmes. For instance, there was a trend towards higher intention in PICANet; this highly specialised audit has a relatively small number of participating sites and may therefore represent a more cohesive, engaged, and responsive network compared with other NCAs. By comparison, the NCABT services a diverse range of topics and clinical settings. A consequence could be differing levels of familiarity with the audit standard selected for each NCA, its credibility, or perceived difficulty of achieving the standard. This is supported by the finding that comprehension and user experience varied less by NCA.

With the exceptions of MINAP and NCABT, intention was generally higher for clinical than managerial, audit, or administrative roles. This is consistent with an earlier modelling experiment evaluating audit and feedback, which found that changes in simulated behaviour were mediated through perceived behavioural control [7]. In our study, clinicians may have perceived greater control over their ability to implement audit standards than those in other roles.

### Strengths and limitations

Previous modelling studies have largely evaluated how feedback in general affects cognitions, but not the effects of individual feedback components [7, 27, 28]. Our fractional factorial design provides information on the effects of both individual and combined modifications and their interactions, demonstrating a rigorous approach for developing multicomponent interventions. Our analysis populations exceeded our sample size requirement of 500 participants, providing over 90% power to detect small to moderate main and interaction effects for each modification. Our use of effect coding also ensured equal power to detect main and interaction effects of the same size. The five NCAs provided diversity in audit methods, topics, and targeted recipients, thereby increasing confidence that the effects we found across NCAs are relevant to a wider range of audit programmes.

Five main study limitations concern the design and “dose’ of the online feedback modifications. First, we selected feedback modifications amenable to online experimentation and which could be operationalised with reasonable fidelity to the original suggestions for effective feedback. Nevertheless, where anticipated effects were not detected, we must consider whether the online feedback modifications were optimised to deliver a sufficient “dose” to influence participant responses and how these could be strengthened in future online experiments or “real-world” pragmatic trials. One case in point is *multimodal feedback*; whilst the Cochrane review indicated that feedback may be more effective when it combines both written and verbal information [1], we operationalised this modification by adding graphical to textual information. The intervention dose may also have been reduced by limited duration of exposure. We originally estimated a completion time of 20–25 min for the audit excerpt and survey; however, participants spent a much lower median time of just over a minute on audit excerpts and less than 5 min on the experiment overall. Whilst these short durations reflect limited engagement, it is uncertain how long feedback recipients would typically spend examining feedback in actual practice settings; it may be relatively brief given competing demands for attention. Therefore, this aspect of our experiment may have reasonable external validity given that much NCA feedback is delivered electronically.

Second, we set out to design a screening experiment which would be relatively sensitive in detecting changes in proximal outcomes of behaviour change, specifically intention to enact audit standards. We would expect some attenuation of effects on intention when the feedback modifications are applied in “real-world” practice, largely because of numerous post-intentional influences on practice (e.g. time and resource constraints). Furthermore, we had anticipated that outcomes measuring intentions would exhibit skew towards higher intention, partly due to social desirability bias. We attempted to neutralise some of this bias by offering statements which recognised that participants would have competing priorities in normal practice. However, the general skewness of outcomes towards higher intentions imposed a ceiling effect on our ability to detect change.

It is worth considering whether intention is the most appropriate primary outcome to use in screening experiments of audit and feedback. CP-FIT hypothesises that several factors, both upstream and downstream to intention, affect the ability of feedback to change clinical behaviour [26]. Upstream influences include interaction with and perception and verification of feedback data. For example, we found that *reducing cognitive load* improved comprehension of data and increased intention to bring audit findings to the attention of colleagues when accompanied by *multimodal feedback*. Therefore, any future experiments could use a wider range of outcomes to reflect different aspects of the whole audit and feedback cycle.

Third, we noted that 11.3% of participants (most commonly managers) dropped out of the experiment prior to questionnaire completion. This suggests a modest degree of self-selection, so that those who completed the experiment might have perceived the experiment or the feedback as more relevant to their roles than those who did not.

Fourth, the integrity of the experiment was threatened by a significant number of duplicative responses. Designing our experiment to maintain participant anonymity of responses meant we could not identify the duplicative responses within experiment data. We therefore minimised the impact of duplicative responses by removing all 603 (49%) responses over the affected period to ensure the primary analysis only included genuine, independent responses. This approach simultaneously discarded unidentifiable genuine responses, representing a waste of research resources and participant time. We conducted sensitivity analyses, excluding only participants who spent less than 20 s completing the experiment questionnaire. This resulted in far fewer exclusions (280, 23%) and a greater proportion of participants included from general practice. Sensitivity analysis of the primary outcome largely supported the modification effects identified. However, we also identified additional effects not detected in the primary analysis, in part due to increased sample size but also due to differences between the two groups of study participants.

Finally, it is noted that any significant effects discussed could be due all or in part to aliasing (designed confounding) (Additional file 1), although this is considered unlikely based on the sparsity of effects principle.

### Implications for practice and research

Our screening experiment aimed to identify single and combined feedback modifications worthy of further real-world trial evaluation. We detected promising signals of effects on intentions for certain combinations of feedback modification and mixed effects of single and combined modifications on a range of proximal intentions, comprehension, and user experience. Although we would be cautious in generalising from an online experiment, we highlight findings with implications for audit programme design and delivery.

We observed potentially important differences between NCAs for intention to enact the audit standards used in the experiment. Further work should explore which aspects of the audit standards, audit organisation, or targeted recipients account for these variations. Our findings suggest a need for national audits to explicitly review the strengths and weaknesses of their whole audit cycles to identify priorities for change. Clinical recipients were more likely to report higher intention than managerial, administrative, and audit staff. Audit programmes should consider reviewing how their feedback is disseminated to staff who are most likely to be able to act on it, particularly clarifying expectations and goals for managers.

The varying interactions between feedback modifications we observed suggest that audit programmes cannot presume that all proposed enhancements to feedback are additive and highlight the need to explicitly consider how different features of feedback might fit and act together, synergistically or antagonistically. As audit and feedback developers are faced with making design decisions on what to include in their feedback interventions, we make specific suggestions based on modification effects supported by good or consistent evidence from the combined analysis of five NCAs:

- Using a comparator aiming to reinforce desired behaviour change (*effective comparators*), which shows recipient performance against the top quarter of performers compared to showing a comparison against overall mean performance, may reduce how easily participants understand audit results and their overall user experience unless accompanied by short, actionable messages with progressive disclosure of additional information (*optional detail*).
- Combining *optional detail and* a quotation and photograph from a fictional patient describing their experience of care related to the associated audit standard (*patient voice*) may improve recipient experience.
- Combining *multimodal feedback* with *optional detail* may reduce intentions to implement audit standards and set goals, comprehension, and recipient experience.
- Many recipients may invest relatively brief time in digesting feedback. *Minimising cognitive load*, by removing distracting detail and additional general text not directly related to the audit standard, may improve comprehension and, when combined with *multimodal feedback*, intention to bring audit findings to the attention of colleagues.

## Conclusion

This randomised fractional factorial screening experiment undertaken across different NCA programmes and healthcare settings has allowed the efficient evaluation of multiple features of feedback interventions. We found that none of six feedback modifications independently increased intention to enact audit standards across clinical and non-clinical recipients. However, potentially important synergistic and antagonistic effects were observed when feedback modifications were combined, as well as dominant influences of audit programme and recipient role. In particular, antagonistic interactions between *multimodal feedback* and *optional detail*, and *cognitive load* and *optional detail*; synergistic interactions between *effective comparators* and *optional detail*, and varying effects of *multimodal feedback* and *cognitive load* across NCAs. Our findings need to be contextualised in the wider theoretical and empirical literature on audit and feedback so that any advice on combining feedback modifications in “real-world” evaluations is based upon those interactions which have the strongest evidence.

## Supplementary Information

**Additional file 1.** Experimental Design.

**Additional file 2.** Populations.

**Additional file 3.** Primary analyses modification effects.

**Additional file 4.** Sensitivity analysis.

## Data Availability

The datasets generated and analysed during the current study are available from the corresponding author on reasonable request.

## References

[1] Ivers N, Jamtvedt G, Flottorp S, Young JM, Odgaard-Jensen J, French SD (2012). Audit and feedback: effects on professional practice and healthcare outcomes. Cochrane Database Syst Rev.

[2] Foy R, Skrypak M, Alderson S, Ivers NM, McInerney B, Stoddart J (2020). Revitalising audit and feedback to improve patient care. BMJ..

[3] Brehaut JC, Colquhoun HL, Eva KW, Carroll K, Sales A, Michie S (2016). Practice feedback interventions: 15 suggestions for optimizing effectiveness. Ann Intern Med.

[4] Foy R, Eccles M, Jamtvedt G, Young J, Grimshaw J, Baker R (2005). What do we know about how to do audit and feedback? Pitfalls in applying evidence from a systematic review. BMC Health Serv Res.

[5] Chakraborty B, Collins LM, Strecher VJ, Murphy SA (2009). Developing multicomponent interventions using fractional factorial designs. Stat Med.

[6] Collins LM, Kugler KC. Optimization of Behavioral, Biobehavioral, and Biomedical Interventions. New York: Springer Publishing; 2018.

[7] Bonetti D, Eccles M, Johnston M, Steen N, Grimshaw J, Baker R (2005). Guiding the design and selection of interventions to influence the implementation of evidence-based practice: an experimental simulation of a complex intervention trial. Soc Sci Med.

[8] Eccles MP, Francis J, Foy R, Johnston M, Bamford C, Grimshaw JM (2009). Improving professional practice in the disclosure of a diagnosis of dementia: a modeling experiment to evaluate a theory-based intervention. Int J Behav Med.

[9] Willis TAW-HA, Weller A, Alderson SL, Wilson S, Walwyn R, Wood S, et al. Interventions to optimise the outputs of national clinical audits to improve the quality of health care: a multi-method study including RCT. Health Soc Care Deliv Res. 2022; (in press);10 (XX). 35767668

[10] Schulz KF, Altman DG, Moher DJT (2010). CONSORT 2010 statement: updated guidelines for reporting parallel group randomised trials. Trials.

[11] Herrett E, Smeeth L, Walker L, Weston C, Heart MAGJ. The myocardial ischaemia national audit project (MINAP). 2010;96(16):1264–7. 10.1136/hrt.2009.192328 PMC3505836 20659944

[12] Willis TAW-HA, Weller A, Alderson SL, Wilson S, Walwyn R, Wood S, et al. Interventions to optimise the outputs of national clinical audits to improve the quality of health care: a multi-method study including RCT. Health Soc Care Deliv Res. 2022; (in press);10(XX). 35767668

[13] Eccles MP, Hrisos S, Francis J, Kaner EF, Dickinson HO, Beyer F (2006). Do self-reported intentions predict clinicians’ behaviour: a systematic review. Implement Sci.

[14] Francis J, Eccles MP, Johnston M, Walker A, Grimshaw JM, Foy R (2004). Constructing questionnaires based on the theory of planned behaviour: a manual for health services researchers.

[15] Kortteisto T, Kaila M, Komulainen J, Mäntyranta T, Rissanen P (2010). Healthcare professionals’ intentions to use clinical guidelines: a survey using the theory of planned behaviour. Implement Sci.

[16] Presseau J, Johnston M, Heponiemi T, Elovainio M, Francis JJ, Eccles MP (2014). Reflective and automatic processes in health care professional behaviour: a dual process model tested across multiple behaviours. Ann Behav Med.

[17] Elling S, Lentz L, De Jong M (2007). Website evaluation questionnaire: development of a research-based tool for evaluating informational websites. International conference on electronic government.

[18] Borsci S, Federici S, Bacci S, Gnaldi M, Bartolucci F (2015). Assessing user satisfaction in the era of user experience: comparison of the SUS, UMUX, and UMUX-LITE as a function of product experience. Int J Hum-Comput Interact.

[19] Lewis JR, Utesch BS, Maher DE, editors. UMUX-LITE: when there’s no time for the SUS. In: Proceedings of the SIGCHI conference on human factors in computing systems. 2013.

[20] Lewis JR, Utesch BS, Maher DE (2015). Measuring perceived usability: the SUS, UMUX-LITE, and AltUsability. Int J Hum-Comput Interact.

[21] Box G, Hunter WC, Stuart JC (1978). Statistics for experimenters: an introduction to design, data analysis and model building.

[22] Wu CJ, Hamada MS (2011). Experiments: planning, analysis, and optimization.

[23] Dean A, Voss D, Draguljić D. Design and analysis of experiments: Springer; 1999.

[24] Van Buuren S, Brand JP, Groothuis-Oudshoorn CG, Rubin DB (2006). Fully conditional specification in multivariate imputation. J Stat Comput Simul.

[25] Rubin DB (1976). Inference and missing data. Biometrika..

[26] Brown B, Gude WT, Blakeman T, van der Veer SN, Ivers N, Francis JJ (2019). Clinical performance feedback intervention theory (CP-FIT): a new theory for designing, implementing, and evaluating feedback in health care based on a systematic review and meta-synthesis of qualitative research. Implement Sci.

[27] Gude WT, Roos-Blom M-J, van der Veer SN, Dongelmans DA, de Jonge E, Francis JJ (2018). Health professionals’ perceptions about their clinical performance and the influence of audit and feedback on their intentions to improve practice: a theory-based study in Dutch intensive care units. Implement Sci.

[28] Gude WT, van Engen-Verheul MM, van der Veer SN, de Keizer NF, Peek N (2017). How does audit and feedback influence intentions of health professionals to improve practice? A laboratory experiment and field study in cardiac rehabilitation. BMJ Qual Saf.

