# Supplementary material for: A randomised fractional factorial screening experiment to predict effective features of audit and feedback
Source: Implement Sci. 2022 May 26;17:34. doi: 10.1186/s13012-022-01208-5 (PMC9137082; doi:10.1186/s13012-022-01208-5)
Supplement: Supplementary file 1 — Additional file 1. Experimental Design. [file 13012_2022_1208_MOESM1_ESM.docx]

# Additional file 1: Experimental Design

Table A1.1 Selected Fractional Factorial Design $\boldsymbol{2}_{\boldsymbol{VI}}^{\boldsymbol{6-1}}$

| **Blocking factor^a^** | **Combination ID** | **Number of Mods “on”** | **Mod/s On** | **Modification ON (+1) or OFF (-1)** | | | | | |
| --- | --- | --- | --- | --- | --- | --- | --- | --- | --- |
|  |  |  |  | **A** | **B** | **C** | **D** | **E** | **F^b^** |
| B1 | C01 | 0 | (1) AllOff | -1 | -1 | -1 | -1 | -1 | -1 |
| B1 | C02 | 2 | *de* | -1 | -1 | -1 | 1 | 1 | -1 |
| B1 | C03 | 2 | *ce* | -1 | -1 | 1 | -1 | 1 | -1 |
| B1 | C04 | 2 | *cd* | -1 | -1 | 1 | 1 | -1 | -1 |
| B1 | C05 | 2 | *bf* | -1 | 1 | -1 | -1 | -1 | 1 |
| B1 | C06 | 4 | *bdef* | -1 | 1 | -1 | 1 | 1 | 1 |
| B1 | C07 | 4 | *bcef* | -1 | 1 | 1 | -1 | 1 | 1 |
| B1 | C08 | 4 | *bcdf* | -1 | 1 | 1 | 1 | -1 | 1 |
| B1 | C09 | 2 | *af* | 1 | -1 | -1 | -1 | -1 | 1 |
| B1 | C10 | 4 | *adef* | 1 | -1 | -1 | 1 | 1 | 1 |
| B1 | C11 | 4 | *acef* | 1 | -1 | 1 | -1 | 1 | 1 |
| B1 | C12 | 4 | *acdf* | 1 | -1 | 1 | 1 | -1 | 1 |
| B1 | C13 | 2 | *ab* | 1 | 1 | -1 | -1 | -1 | -1 |
| B1 | C14 | 4 | *abde* | 1 | 1 | -1 | 1 | 1 | -1 |
| B1 | C15 | 4 | *abce* | 1 | 1 | 1 | -1 | 1 | -1 |
| B1 | C16 | 4 | *abcd* | 1 | 1 | 1 | 1 | -1 | -1 |
| B2 | C17 | 2 | *ef* | -1 | -1 | -1 | -1 | 1 | 1 |
| B2 | C18 | 2 | *df* | -1 | -1 | -1 | 1 | -1 | 1 |
| B2 | C19 | 2 | *cf* | -1 | -1 | 1 | -1 | -1 | 1 |
| B2 | C20 | 4 | *cdef* | -1 | -1 | 1 | 1 | 1 | 1 |
| B2 | C21 | 2 | *be* | -1 | 1 | -1 | -1 | 1 | -1 |
| B2 | C22 | 2 | *bd* | -1 | 1 | -1 | 1 | -1 | -1 |
| B2 | C23 | 2 | *bc* | -1 | 1 | 1 | -1 | -1 | -1 |
| B2 | C24 | 4 | *bcde* | -1 | 1 | 1 | 1 | 1 | -1 |
| B2 | C25 | 2 | *ae* | 1 | -1 | -1 | -1 | 1 | -1 |
| B2 | C26 | 2 | *ad* | 1 | -1 | -1 | 1 | -1 | -1 |
| B2 | C27 | 2 | *ac* | 1 | -1 | 1 | -1 | -1 | -1 |
| B2 | C28 | 4 | *acde* | 1 | -1 | 1 | 1 | 1 | -1 |
| B2 | C29 | 4 | *abef* | 1 | 1 | -1 | -1 | 1 | 1 |
| B2 | C30 | 4 | *abdf* | 1 | 1 | -1 | 1 | -1 | 1 |
| B2 | C31 | 4 | *abcf* | 1 | 1 | 1 | -1 | -1 | 1 |
| B2 | C32 | 6 | *abcdef* | 1 | 1 | 1 | 1 | 1 | 1 |

*^a^ Block Pseudo-factor Confounding Rules: [B1] = ABF = CDE. In this block design the two blocks are made up of the 2^4^ factorial design for modifications A, B, C, D. ^b^ Defining relation F=ABCDE*

Table A1.2 Alias Structure of the Selected Fractional Factorial Design $\boldsymbol{2}_{\boldsymbol{VI}}^{\boldsymbol{6-1}}$

| **Alias structure** |
| --- |
| 0=ABCDEF, A=BCDEF, B=ACDEF, C=ABDEF, D=ABCEF, E=ABCDF, F=ABCDE |
| AB=CDEF, AC=BDEF, AD=BCEF, AE=BCDF, AF=BCDE, BC=ADEF, BD=ACEF, BE=ACDF, BF=ACDE, CD=ABEF, CE=ABDF, CF=ABDE, DE=ABCF, DF=ABCE, EF=ABCD |
| ABC=DEF, ABD=CEF, ABE=CDF, [B1]=ABF=CDE, ACD=BEF, ACE=BDF, ACF=BDE, ADE=BCF, ADF=BCE, AEF=BCD |

**Figure A.1 ENACT experiment audit report - NDA participant allocated to modification combination C19 “CF”***


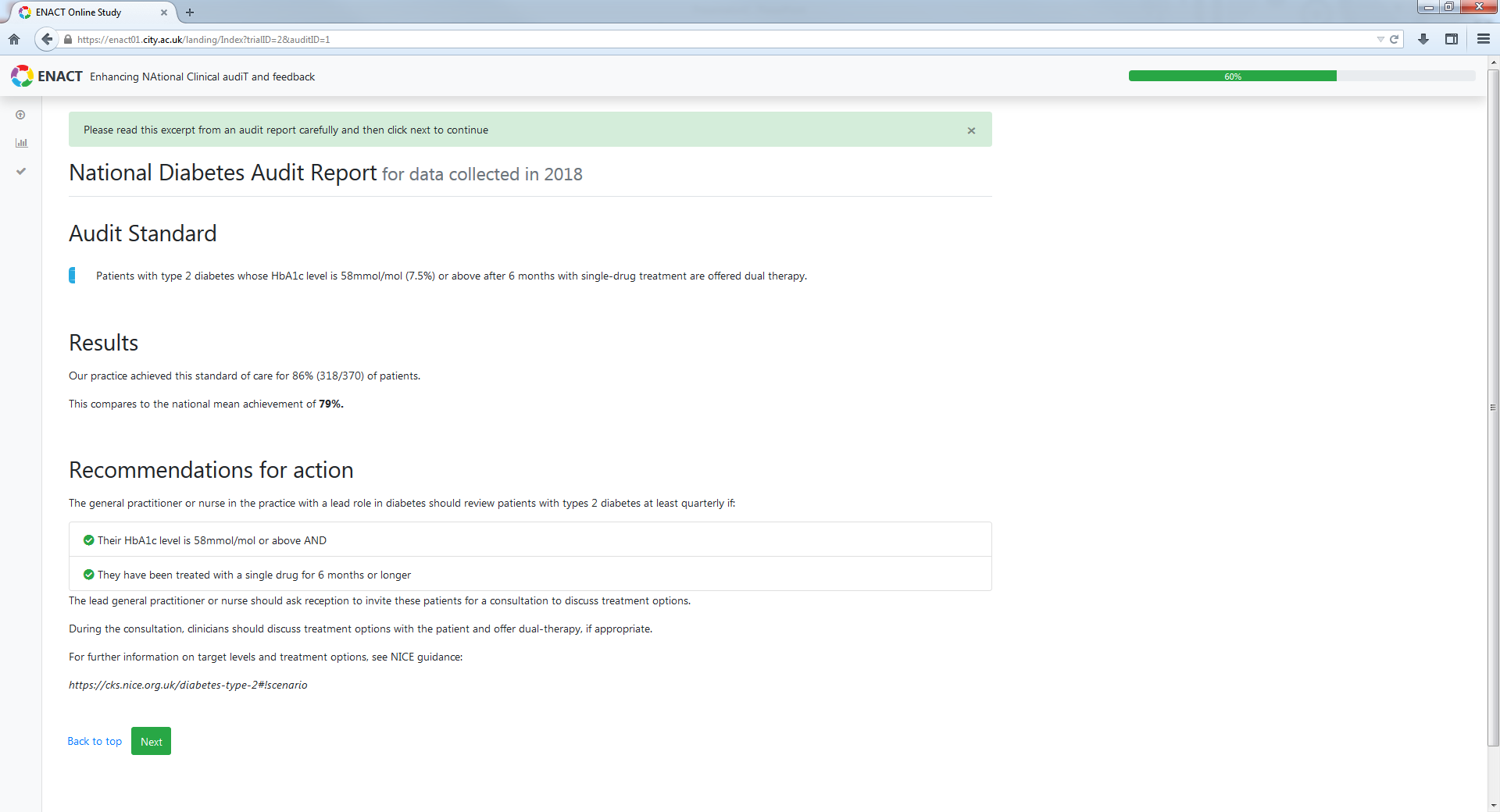
*Modifications ON: Specific actions (C) and Reduced cognitive load (F). Modifications OFF: Effective comparators (A), Multimodal feedback (B), Optional detail (D), Patient voice (E).
