# Supplementary material for: A randomised fractional factorial screening experiment to predict effective features of audit and feedback
Source: Implement Sci. 2022 May 26;17:34. doi: 10.1186/s13012-022-01208-5 (PMC9137082; doi:10.1186/s13012-022-01208-5)
Supplement: Supplementary file 2 — Additional file 2. Populations. [file 13012_2022_1208_MOESM2_ESM.docx]

# Additional file 2: Populations

*
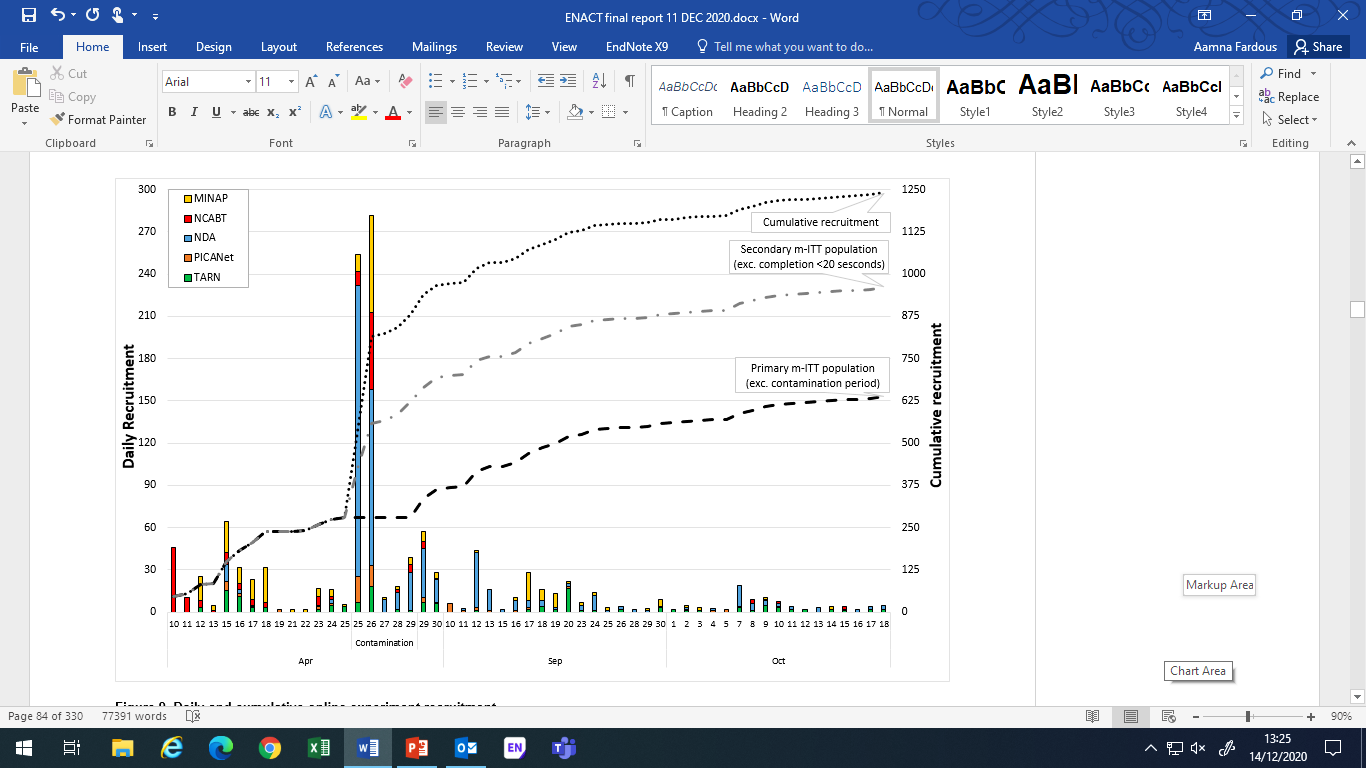
*

Figure A2.1. Daily and cumulative online experiment recruitment

Table A2.1. Time spent on questionnaire

|  | **Completion during the contamination period?** | | |
| --- | --- | --- | --- |
|  | **Yes (n=597)** | **No (n=629)** | **Total (n=1226)** |
| **Time on questionnaire (seconds)** |  |  |  |
| N – Total completing questionnaire | 547 | 566 | 1113 |
| Median (Range) | 31.0 (3.5, 19783.0) | 159.0 (2.5, 16320.0) | 113.5 (2.5, 19783.0) |
| Interquartile Range | (13.0, 139.0) | (97.5, 255.5) | (19.0, 205.0) |
| **>20s on questionnaire** |  |  |  |
| Yes | 288 (52.7%) | 545 (96.3%) | 833 (74.8%) |
| No | 259 (47.3%) | 21 (3.7%) | 280 (25.2%) |

| (a) | 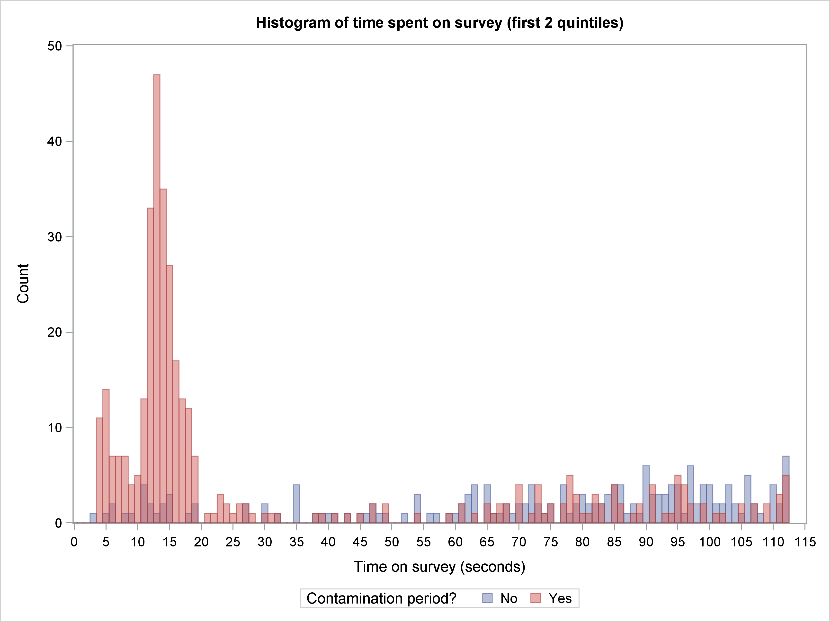 |
| --- | --- |
| (b) | 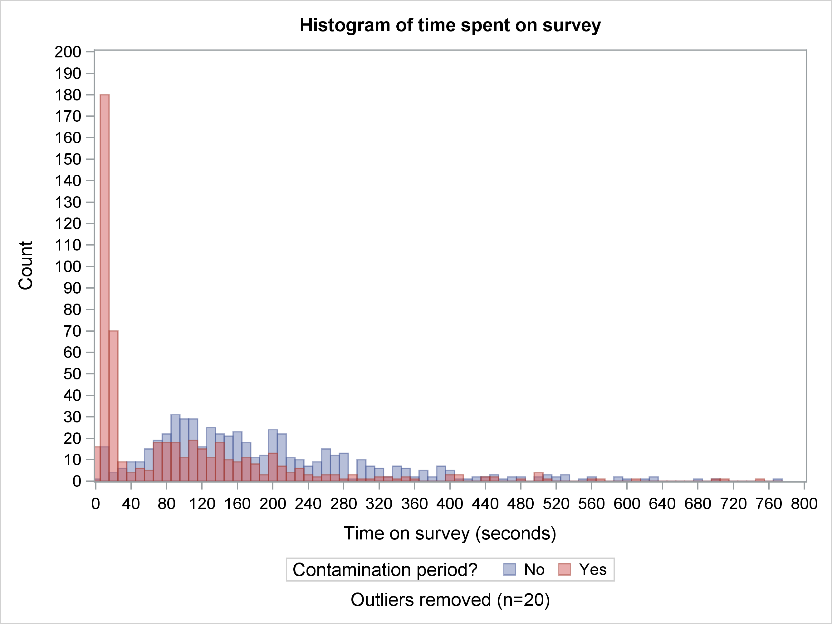 |

**Figure A2.2. Histogram of time spent on questionnaire survey: (a) first 2 quintiles; (b) overall – excluding largest 20 outliers**

Table A2.2. Number of participants in each population by audit

|  | **MINAP** | **NCABT** | **NDA** | **PICANet** | **TARN** | **Total** |
| --- | --- | --- | --- | --- | --- | --- |
| **Participant in primary**  **modified ITT population?** | | |  |  |  |  |
| Yes | 178 (66.7%) | 102 (58.3%) | 204 (34.9%) | 36 (52.2%) | 118 (80.8%) | 638 (51.4%) |
| No - Recruited during contamination period | 89 (33.3%) | 73 (41.7%) | 380 (65.1%) | 33 (47.8%) | 28 (19.2%) | 603 (48.6%) |
| **Participant in secondary**  **modified ITT population?** | | |  |  |  |  |
| Yes | 211 (79.0%) | 129 (73.7%) | 457 (78.3%) | 34 (49.3%) | 130 (89.0%) | 961 (77.4%) |
| No - Completed questionnaire in <20 seconds | 56 (21.0%) | 46 (26.3%) | 127 (21.7%) | 35 (50.7%) | 16 (11.0%) | 280 (22.6%) |
| **Total Randomisations** | **267 (100%)** | **175 (100%)** | **584 (100%)** | **69 (100%)** | **146 (100%)** | **1241 (100%)** |

## Participant characteristics

The primary modified ITT population comprised a greater proportion of participants from hospital trusts (64.9%) and a reduced proportion of participants from general practice (29.6%) compared to all randomisations (41.3%, 45% respectively) and participants in the secondary modified ITT population (49.8%, 44.8% respectively). This was largely because the contamination period coincided with the distribution of the experiment for the NDA, for which general practices were the main recipients. A similar but less distinct pattern was observed by role, with a reduction in the proportion of managers in the primary modified ITT population (27.3%) compared to all randomisations (34.7%) and participants in the secondary modified ITT population (36.2%).

## Randomisation

There was greater variability in the number of participants allocated to each of the 32 modification combinations within the secondary modified ITT population, however the proportion of participants with each version of modifications (ON or OFF) was relatively stable across populations.

## Experiment completion

A similar distribution of characteristics and completion rates across modifications was observed for each population.

## Outcomes

Across the populations, responses for the primary outcome were more uniform for the primary modified ITT population, with slightly increased skew in the secondary modified ITT population.

|  |
| --- |
|  |
|  |

**Figure A2.3. Randomly allocated modification combination by NCA and population**

|  |
| --- |
|  |
|  |

**Figure A2.4. Randomly allocated modifications by NCA and population**
