# Supplementary material for: A randomised fractional factorial screening experiment to predict effective features of audit and feedback
Source: Implement Sci. 2022 May 26;17:34. doi: 10.1186/s13012-022-01208-5 (PMC9137082; doi:10.1186/s13012-022-01208-5)
Supplement: Supplementary file 3 — Additional file 3. Primary analyses modification effects. [file 13012_2022_1208_MOESM3_ESM.docx]

# Additional file 3: Primary analyses modification effects

## This additional file provides supporting figures for the results presented in the main report. Extracts of the text within the main report relevant to each figure are copied following each figure to provide easy navigation between the main results and the additional figures.

## **Stage Two -** Parsimonious models


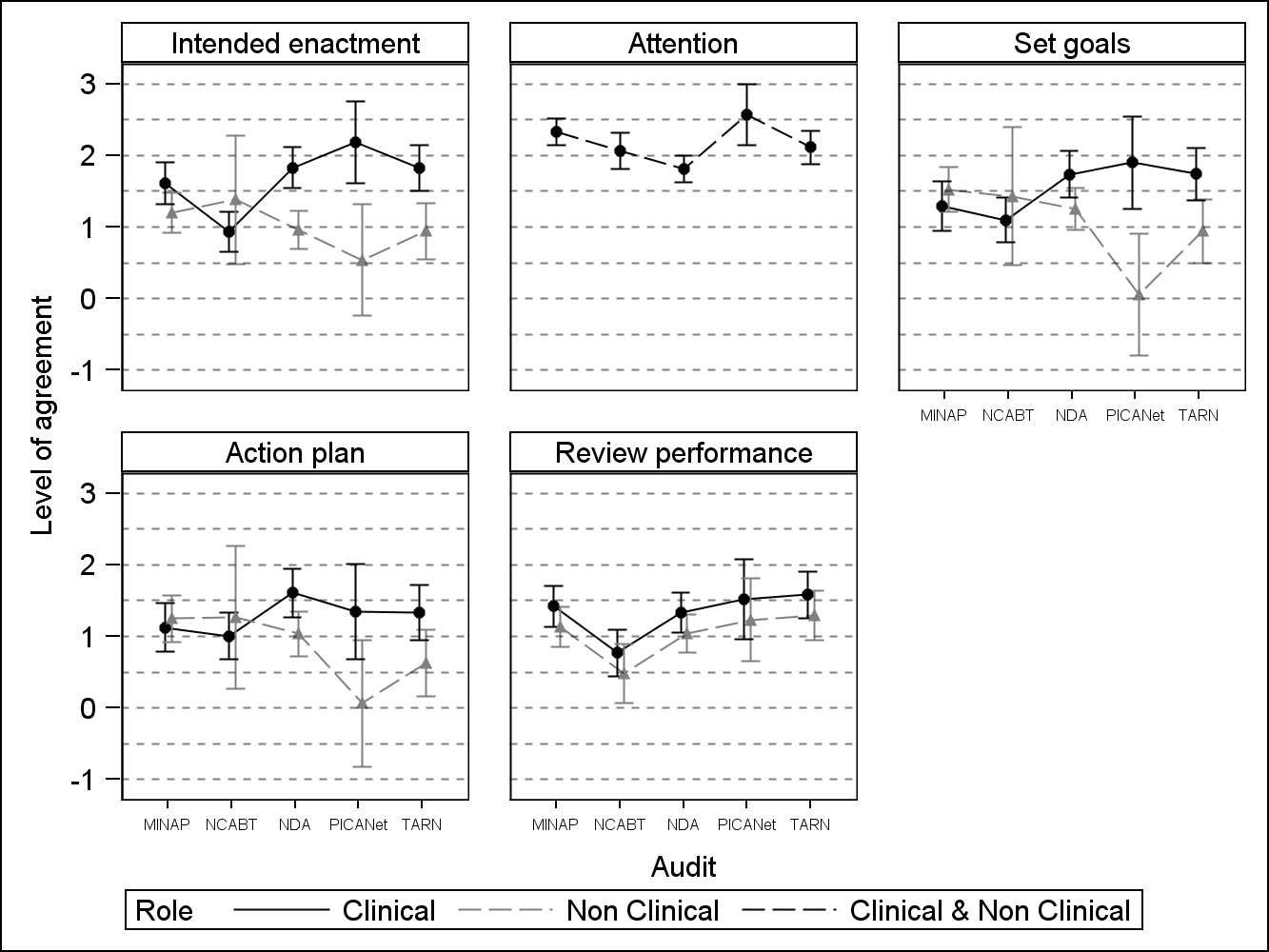


Figure A3.1. Predicted agreement by Audit and Role for primary outcome intended enactment and secondary proximal intention outcomes

“Intention was lower for non-clinical than clinical participants (-0.867, SE=0.200, p<0.001) in the NDA with similar effects observed within MINAP, PICANet and TARN. This effect was not observed within the NCABT: intention was lower for clinical NCABT participants than clinical participants in the NDA (-0.893, SE=0.206, p<0.001) and other NCAs (Additional file 3, Figure A3.1).”

“Audit and role had similar dominant influences on secondary outcomes of proximal intention (Figure A3.1).

Non-clinical (compared to clinical) participants had lower intention to set goals (-0.489, SE=0.226, p=0.030), create an action plan (-0.571, SE=0.235, p=0.015) and review performance (-0.29, SE=0.143, p=0.043). Interactions with audit mitigated this effect within MINAP for intention to set goals (MINAP -0.445, SE=0.245, p=0.069; NonClinical*MINAP, 0.721, SE=0.332, p=0.030) and to set an action plan (MINAP -0.482, SE=0.245, p=0.049; NonClinical*MINAP, 0.695, SE=0.339, p=0.040); and accentuated the effect in PICANet (NonClinical*PICANet, -1.360, SE=0.590, p=0.021) on intention to set goals.

As per the primary outcome, reduced intention within clinical NCABT (compared to clinical NDA) participants also mitigated the difference between roles for intention to set goals (NCABT, -0.638, SE=0.234, p=0.006), to set an action plan (NCABT, -0.603, SE=0.238, p=0.011) and to review performance (NCABT, -0.565, SE=0.213, p=0.008). There was no evidence of a difference in intention to bring the audit to the attention of colleagues according to role, however intention was lowest among participants from the NDA. There was no evidence of a difference in comprehension or user experience according to audit or role.”

| 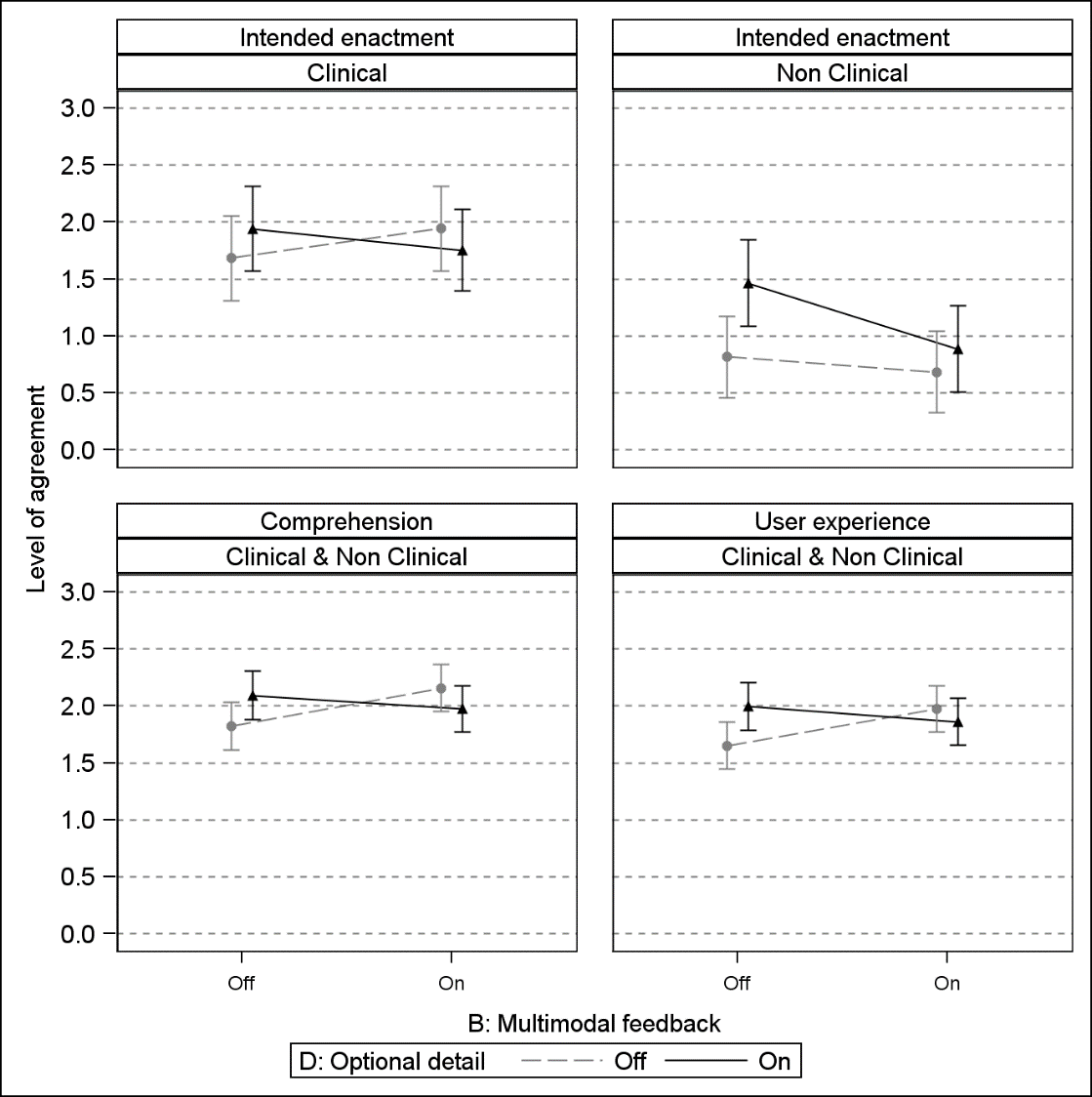  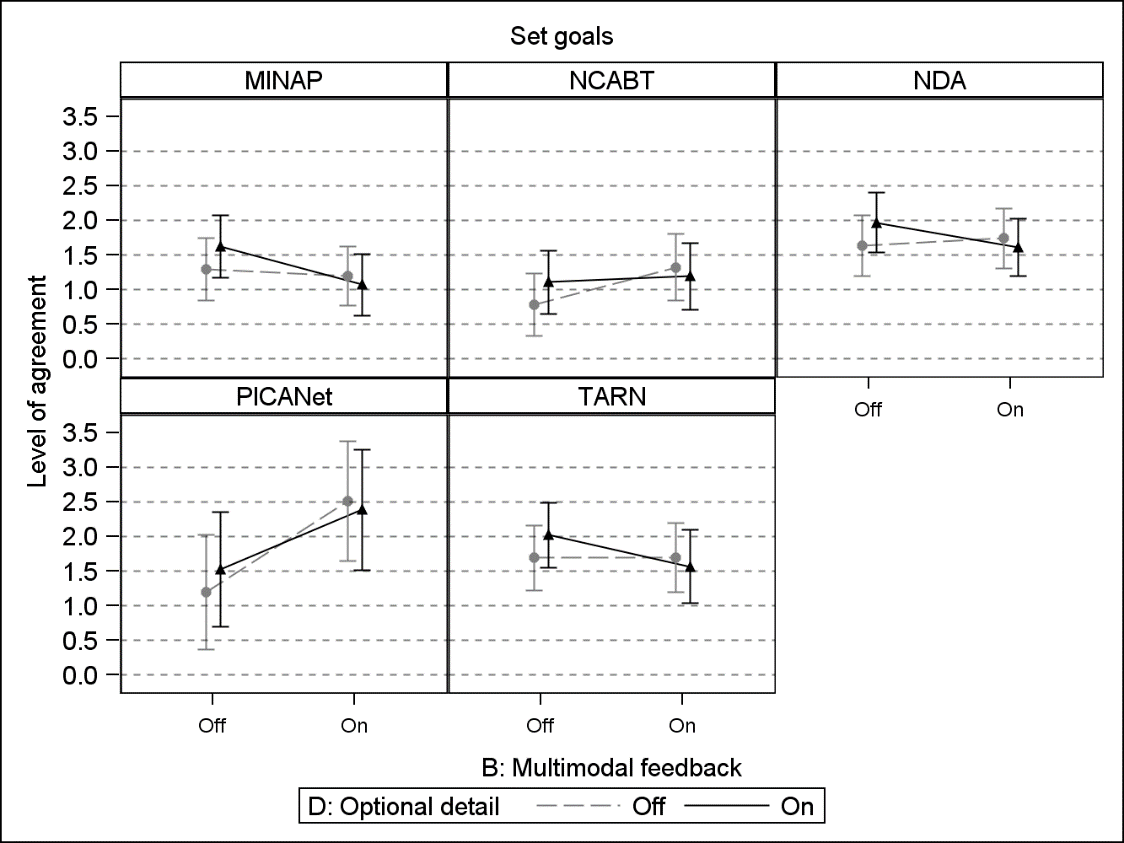 |
| --- |

Figure A3.2. Predicted agreement by multimodal feedback and optional detail (B*D) for outcomes: intended enactment in NDA clinical recipients accounting for B*Role and D*Role interactions; comprehension and user experience in NDA recipients; and intention to set goals accounting for B*audit interaction.

“There was evidence of an antagonistic interaction between multimodal feedback and optional detail (-0.112, SE=0.056, p=0.047); intention was lower when both were applied (or not), and higher when only one or the other was applied (Figure A3.2). . In non-clinical participants there was weak evidence of a negative effect of multimodal feedback (-0.196, SE=0.113, p=0.083), and a positive effect of optional detail (0.195, SE=0.114, p=0.087), with intention optimised when optional detail, not multimodal feedback, was provided.”

“*Modifications multimodal feedback*optional detail.* Alongside the primary outcome, there was evidence (Figure A3.2) of an antagonistic interaction between multimodal feedback and optional detail on secondary outcomes intention to set goals (-0.114, SE=0.062, p=0.067), comprehension (-0.114, SE=0.043, p=0.008) and user experience (-0.115, SE=0.042, p=0.006). Outcomes were generally improved when only one of the modifications was applied; however, intention to set goals was optimised in PICANet participants when multimodal feedback was included and not optional detail (0.607, SE=0.288, p=0.035).”


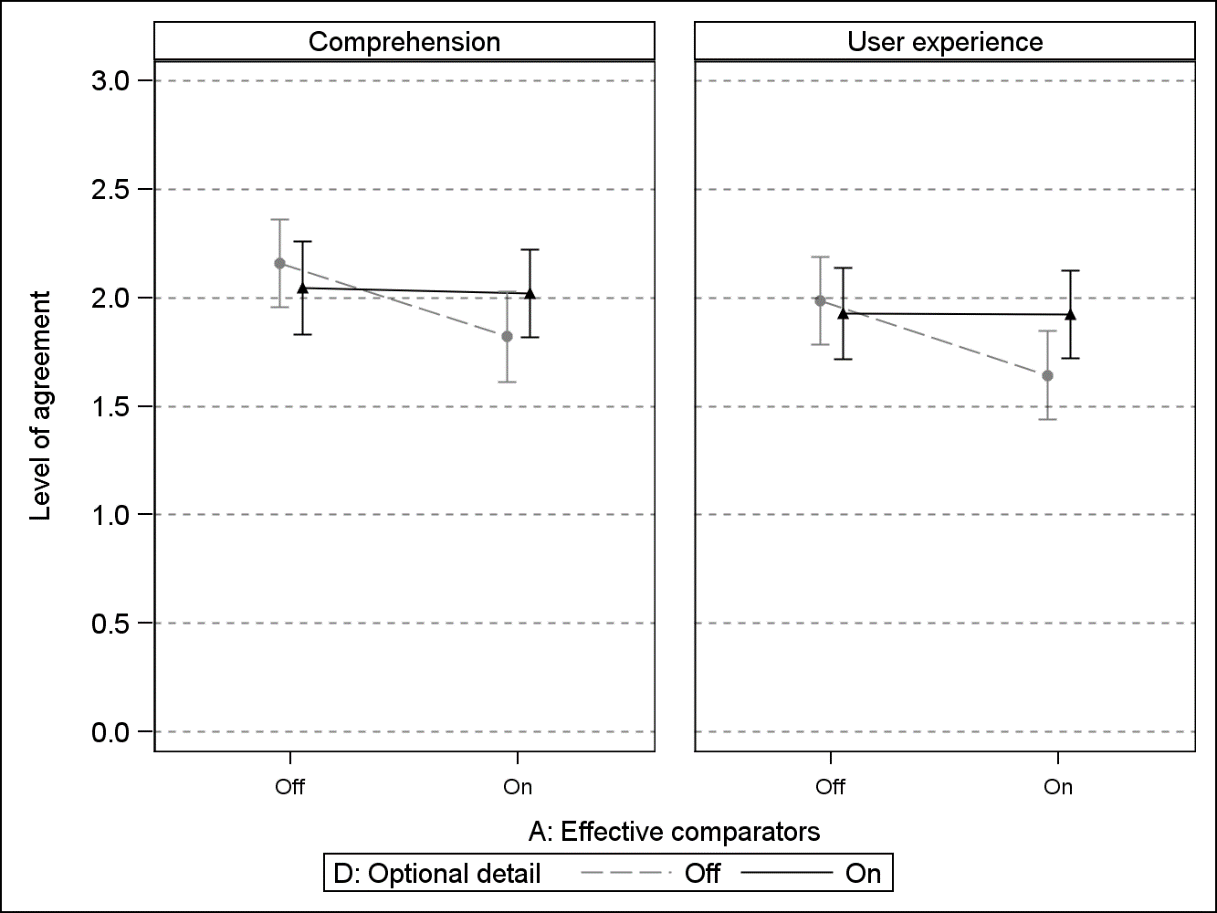


Figure A3.3. Predicted agreement by effective comparators and optional detail (A*D) for comprehension and user experience outcomes in NDA clinical recipients

“*Modifications: effective comparators and effective comparators*optional detail*. An overall average effect of effective comparators reduced how easily participants understood the audit report (-0.091, SE=0.041, p=0.029) and their overall user experience (-0.087, SE=0.041, p=0.036). Synergistic interactions between the comparator and optional detail for these outcomes, comprehension (0.078, SE=0.043, p=0.068) and overall user experience (0.085, SE=0.042, p=0.043), meant the negative comparator effect was not present when optional detail was provided (Figure A3.3).”

| 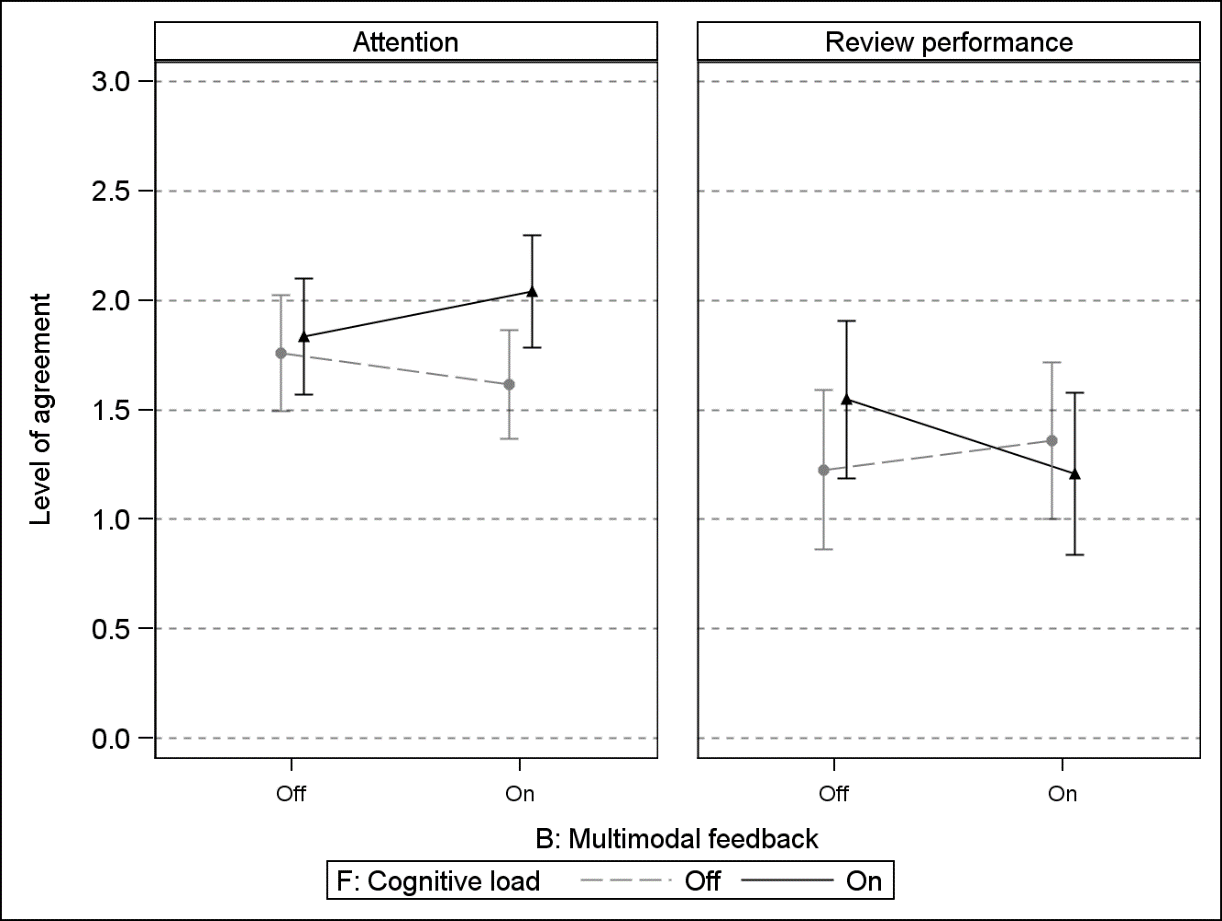  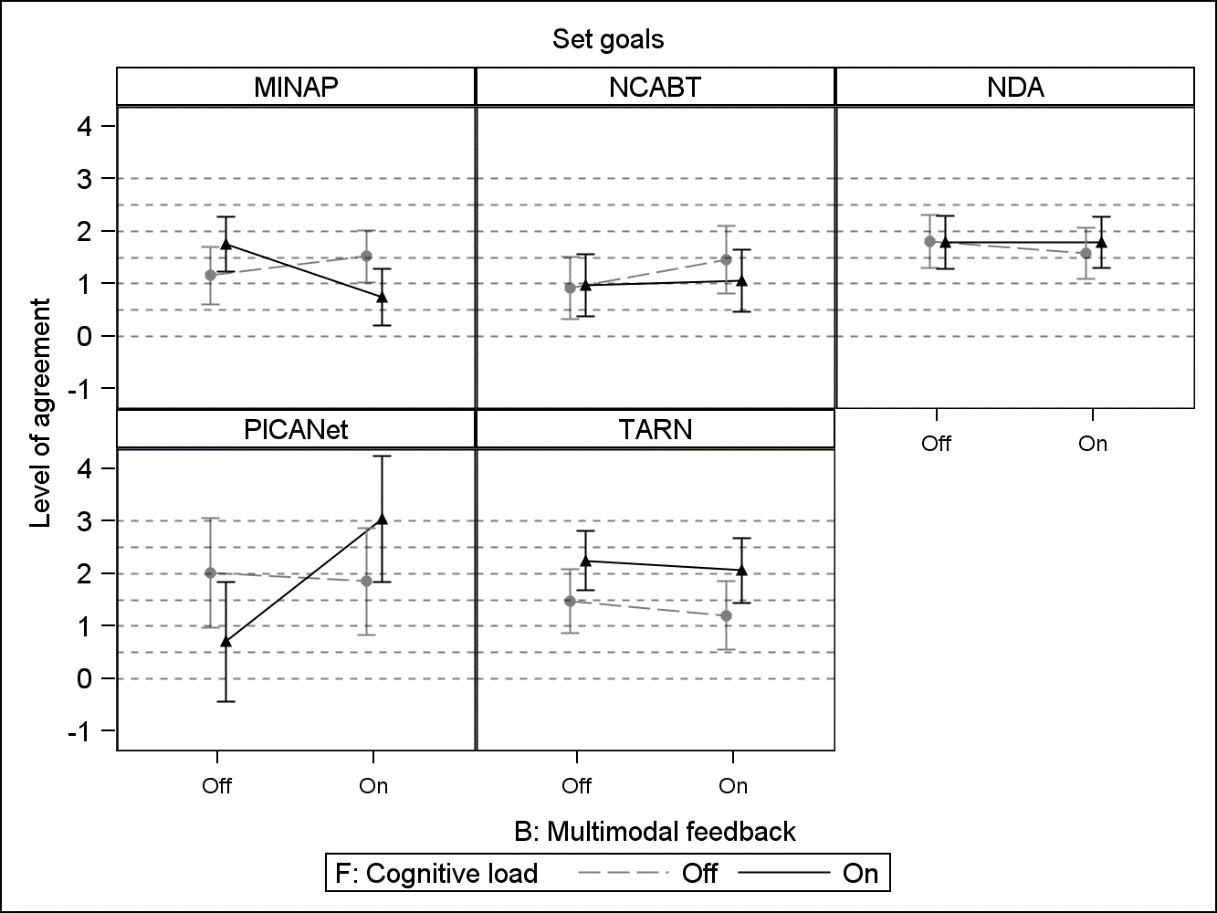 |
| --- |

Figure A3.4. Predicted agreement by multimodal feedback and cognitive load (B*F) for: intention to bring audit report to attention of colleagues and to review performance in NDA clinical recipients; and intention to set goals accounting for B*F*audit interaction in clinical recipients


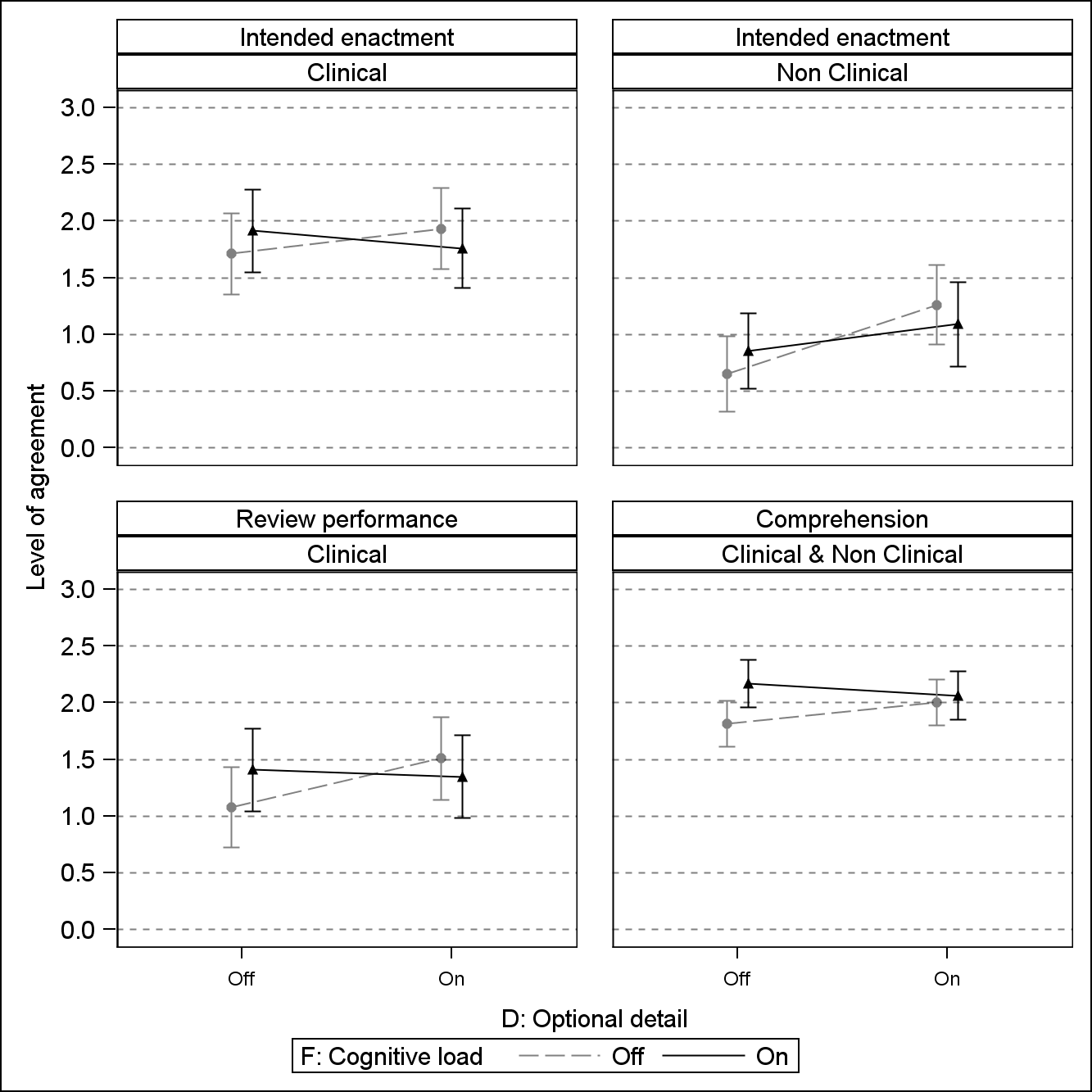
Figure A3.5. Predicted agreement by optional detail and cognitive load (D*F) in the NDA for intended enactment accounting for D*Role interaction; intention to review performance in clinical recipients and comprehension across recipients.

“*Modifications: cognitive load, multimodal feedback*effective comparators, optional detail*effective comparators.* The overall average effect of reducing cognitive load improved intention to bring the report to the attention of colleagues (0.126, SE=0.052, p=0.016, Figure A3.4) and comprehension (0.103, SE=0.042, p=0.014, Figure A3.5). A synergistic interaction between cognitive load and multimodal feedback (0.087, SE=0.051, p=0.089, Figure A3.4), improved intention to bring the report to the attention of colleagues further when multimodal feedback was also provided.”


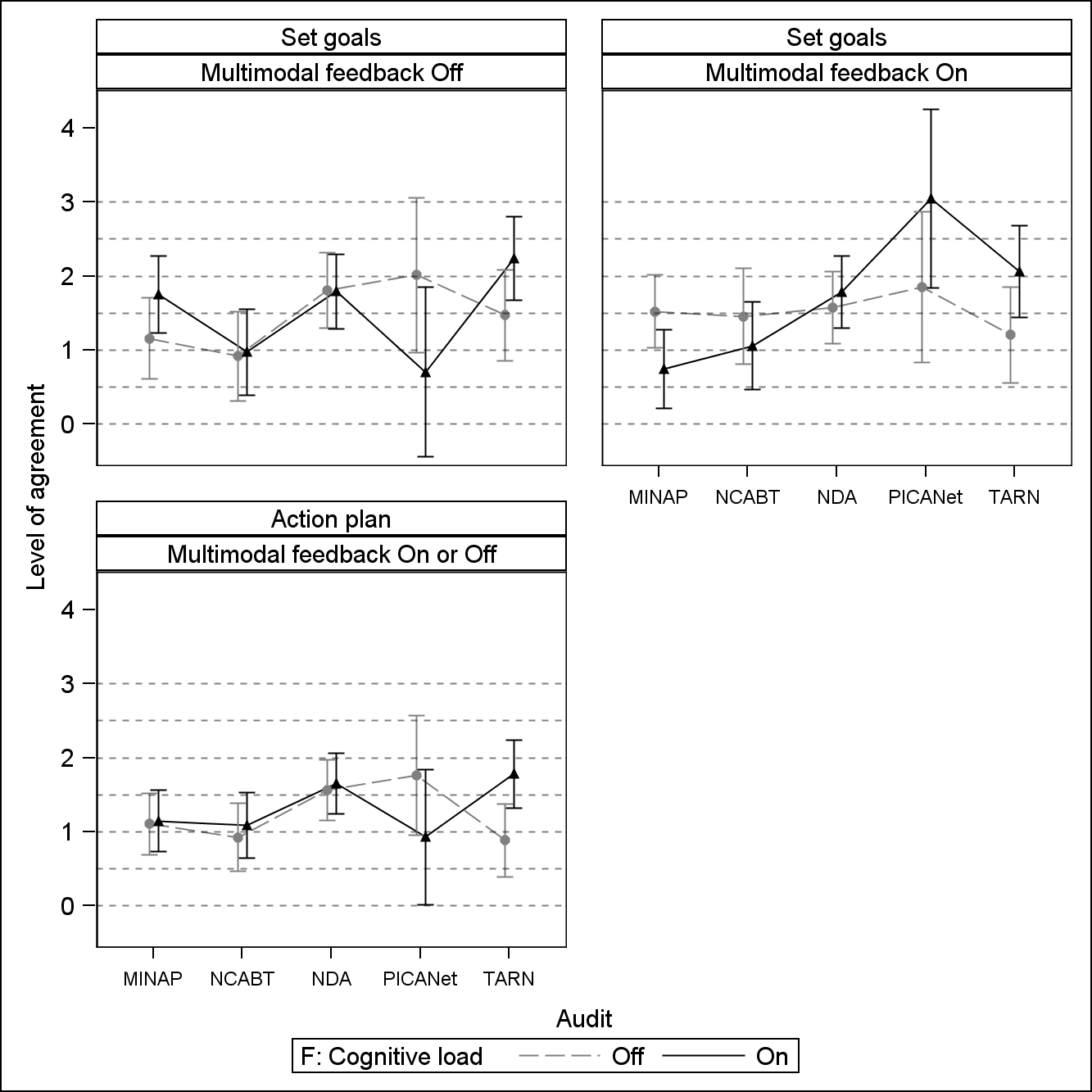
Figure A3.6. Predicted agreement by cognitive load and audit in clinical recipients for intention to set goals accounting for multimodal feedback (B*F), and intention to set an action plan.

“*Modifications: cognitive load, multimodal feedback*effective comparators, optional detail*effective comparators.*

A similar synergistic interaction was detected for PICANet participants on intention to set goals (0.569, SE=0.285, p=0.046, Figure A3.6). The opposite antagonist effect was present for MINAP participants (-0.4, SE=0.162, p=0.013, Figure A3.6) and a weak antagonistic effect was detected for intention to review performance (-0.119, SE=0.069, p=0.085, Figure A3.4) across audits.

In TARN participants, reduced cognitive load also improved intention to set goals (0.357, SE=0.182, p=0.049, Figure A3.4) and an action plan (0.407, SE=0.191, p=0.033, Figure A3.6).

Across all NCAs, reducing cognitive load without providing optional detail improved comprehension (-0.073, SE=0.042, p=0.079, Figure A3.5). A similar antagonistic interaction was detected on the primary outcome (-0.093, SE=0.055, p=0.089, Figure A3.5) and intention to review performance (-0.123, SE=0.066, p=0.065, Figure A3.5).”


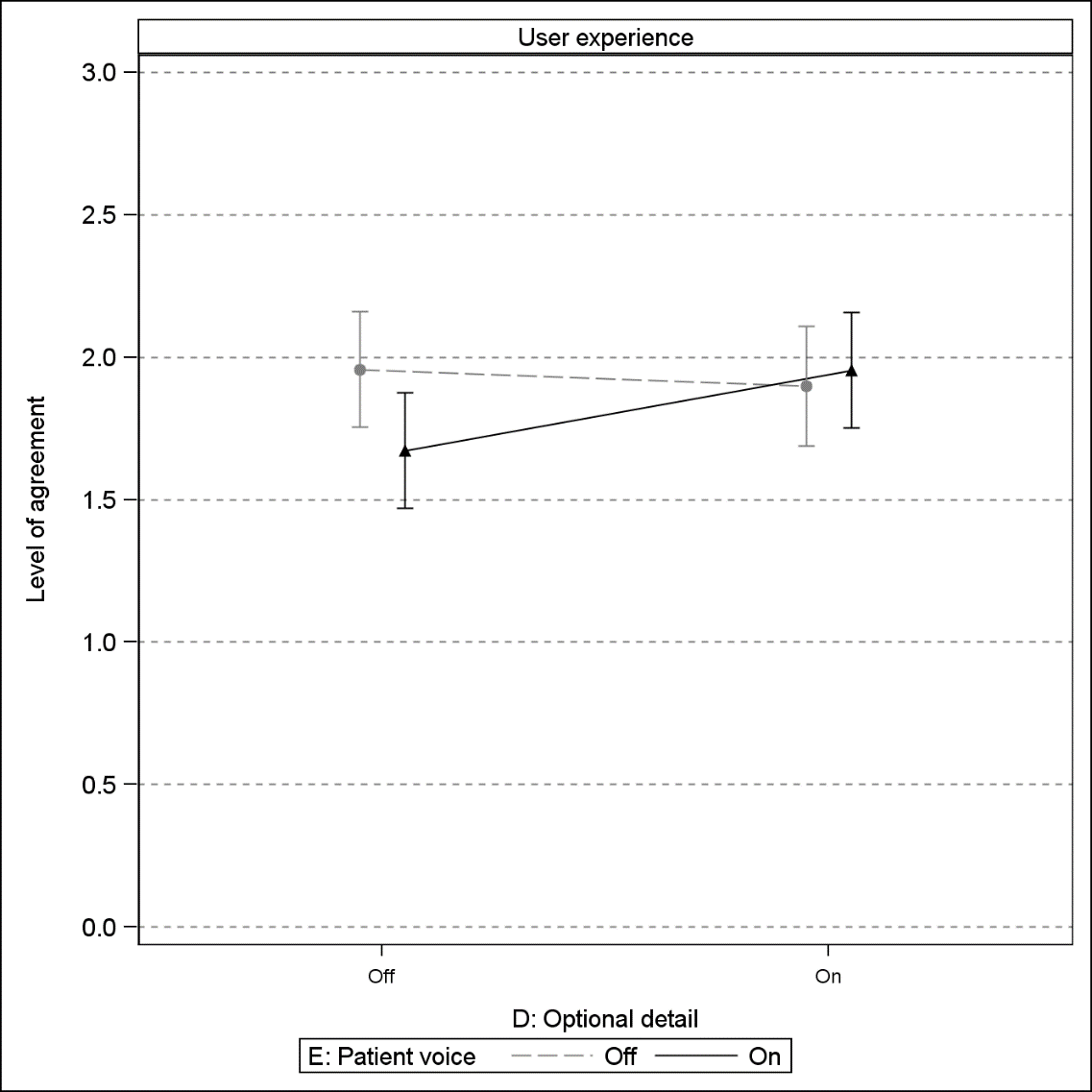


Figure A3.7. Predicted agreement by optional detail and patient voice (D*E) in clinical NDA recipients for user experience.

*“Modifications: optional detail*patient voice.* There was a synergistic interaction between optional detail and patient voice on user experience such that including patient voice without optional detail reduced user experience (0.085, SE=0.041, p=0.039) (Figure A3.7).”

| 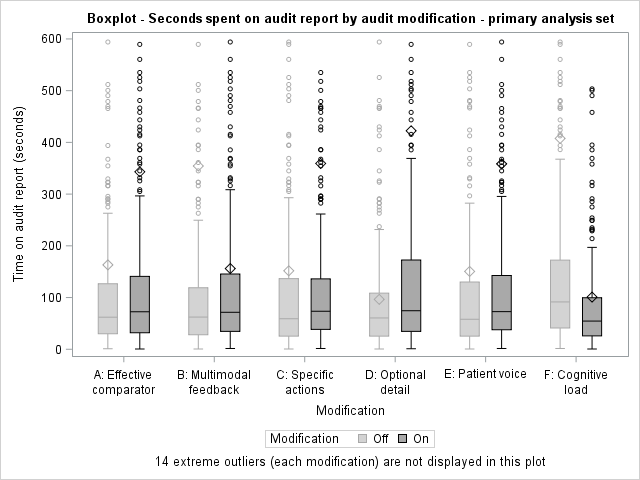 |
| --- |
|  |

Figure A3.8. Boxplot of user engagement - time on audit report - by modifications and audit*

*14 extreme outliers are not displayed

|  |
| --- |
|  |

Figure A3.9. Boxplot of user engagement - clicks on audit report - by modifications and audit*

*5 extreme outliers are not displayed

“User engagement. As anticipated, median time spent on the audit tended to be higher when each of the modifications were on, with the exception of reduced cognitive load (Figure A3.8). We observed the greatest number of clicks on the audit excerpt when optional detail was on compared to off (Figure A3.8).”
