# Supplementary material for: A randomised fractional factorial screening experiment to predict effective features of audit and feedback
Source: Implement Sci. 2022 May 26;17:34. doi: 10.1186/s13012-022-01208-5 (PMC9137082; doi:10.1186/s13012-022-01208-5)
Supplement: Supplementary file 4 — Additional file 4. Sensitivity analysis. [file 13012_2022_1208_MOESM4_ESM.docx]

# Additional file 4: Sensitivity analysis

Table A4.1. Primary outcome: Comparative parameter estimates for the primary and sensitivity analysis

|  | **Primary analysis**  **Primary modified ITT population (N=638,**  **exc. contamination period)** | | | **Sensitivity analysis**  **Secondary modified ITT population (N=833,**  **exc. completers <20 seconds)** | | |
| --- | --- | --- | --- | --- | --- | --- |
| **Parameter** | **Estimate** | **Standard Error** | **p-value** | **Estimate** | **Standard Error** | **p-value \|** |
| **Intercept** | 1.829 | 0.147 | <.001 | 1.898 | 0.110 | <.001 |
| **Block** | 0.090 | 0.056 | 0.107 | 0.037 | 0.045 | 0.411 |
| **Audit** |  |  |  |  |  |  |
| MINAP | -0.211 | 0.211 | 0.317 | -0.258 | 0.179 | 0.150 |
| NCABT | -0.893 | 0.206 | **<.001**** | -1.004 | 0.168 | **<.001***** |
| PICANet | 0.361 | 0.327 | 0.270 | 0.101 | 0.319 | 0.751 |
| TARN | -0.003 | 0.220 | 0.989 | -0.120 | 0.189 | 0.526 |
| **Non-Clinical** (vs Clinical) | -0.867 | 0.200 | **<.001**** | -0.790 | 0.139 | **<.001***** |
| **A:** Effective comparator | -0.038 | 0.056 | 0.498 | 0.125 | 0.066 | 0.060 |
| **B:** Multimodal feedback | 0.018 | 0.073 | 0.807 | 0.049 | 0.064 | 0.442 |
| **C:** Specific actions | 0.082 | 0.056 | 0.141 | 0.033 | 0.045 | 0.462 |
| **D:** Optional detail | 0.017 | 0.074 | 0.816 | 0.059 | 0.045 | 0.193 |
| **E:** Patient voice | 0.078 | 0.055 | 0.161 | 0.073 | 0.045 | 0.105 |
| **F:** Cognitive load | 0.008 | 0.055 | 0.890 | -0.095 | 0.063 | 0.135 |
| **A * B** | -0.011 | 0.055 | 0.844 | -0.004 | 0.045 | 0.932 |
| **A * D** | **--** | **--** | **--** | 0.020 | 0.045 | 0.656 |
| **A * E** | -0.014 | 0.056 | 0.798 | **--** | **--** | **--** |
| **B * D** | -0.112 | 0.056 | **0.047**** | -0.089 | 0.045 | **0.049**** |
| **B * E** | 0.035 | 0.057 | 0.537 | **--** | **--** | **--** |
| **C * F** | 0.093 | 0.055 | 0.090 | 0.071 | 0.045 | 0.114 |
| **D * F** | -0.093 | 0.055 | 0.089 | -0.097 | 0.045 | **0.032**** |
| Non-Clinical * MINAP | 0.453 | 0.290 | 0.117 | 0.258 | 0.237 | 0.278 |
| Non-Clinical * NCABT | 1.312 | 0.518 | **0.011**** | 1.207 | 0.439 | **0.006**** |
| Non-Clinical * PICA | -0.783 | 0.532 | 0.141 | -0.627 | 0.511 | 0.220 |
| Non-Clinical * TARN | -0.017 | 0.331 | 0.959 | 0.054 | 0.277 | 0.846 |
| **A * MINAP** | **--** | **--** | **--** | -0.074 | 0.116 | 0.527 |
| **A * NCABT** | **--** | **--** | **--** | -0.246 | 0.139 | 0.077 |
| **A * PICA** | **--** | **--** | **--** | -0.342 | 0.248 | 0.169 |
| **A * TARN** | **--** | **--** | **--** | -0.302 | 0.135 | **0.026**** |
| **B * Non-Clinical** | -0.196 | 0.113 | 0.083 | -0.151 | 0.091 | 0.095 |
| **D * Non-Clinical** | 0.195 | 0.114 | 0.087 | **--** | **--** | **--** |
| **F * Non-Clinical** | **--** | **--** | **--** | 0.156 | 0.090 | 0.083 |
| **A * B * D = C * E * F** | **--** | **--** | **--** | -0.088 | 0.045 | **0.052**** |
| **A * B * E = C * D * F** | -0.101 | 0.056 | 0.072 | **--** | **--** | **--** |
